# Genetic diversity of pangolin coronaviruses reveals a key immuno-evasive substitution at spike residue 519

**DOI:** 10.1128/jvi.00352-26

**Published:** 2026-06-10

**Authors:** Maximilian Stanley Yo, Yu Kaku, Yusuke Kosugi, Jarel Elgin Tolentino, Daisuke Kuroda, Yunlong Cao, Kei Sato

**Affiliations:** 1 Division of Systems Virology, Department of Microbiology and Immunology, The Institute of Medical Science, The University of Tokyo 515734 , Tokyo, Japan; 2 Department of Computational Biology and Medical Sciences, Graduate School of Frontier Sciences, The University of Tokyo 200705 , Kashiwa, Japan; 3 Department of Pathology, Immunology and Microbiology, Graduate School of Medicine, The University of Tokyo 515734 , Tokyo, Japan; 4 Department of Biosciences, College of Humanities and Sciences, Nihon University 12976, Tokyo, Japan; 5 Biomedical Pioneering Innovation Center (BIOPIC), School of Life Sciences, Peking University 12465, Beijing, China; 6 Changping Laboratory 662243 , Beijing, China; 7 Peking-Tsinghua Center for Life Sciences, Peking University 12465, Beijing, China; 8 International Research Center for Infectious Diseases, The Institute of Medical Science, The University of Tokyo 515734 , Tokyo, Japan; 9 International Vaccine Design Center, The Institute of Medical Science, The University of Tokyo 515734 , Tokyo, Japan; 10 Collaboration Unit for Infection, Joint Research Center for Human Retrovirus infectionn, Kumamoto University 13205, Kumamoto, Japan; 11 MRC-University of Glasgow Centre for Virus Research 155698, Glasgow, United Kingdom; 12 Faculty of Medicine, Chulalongkorn University 26683, Bangkok, Thailand; 13 Programme in Emerging Infectious Diseases, Duke-NUS Medical School 121579, Singapore, Singapore; Cornell University Baker Institute for Animal Health, Ithaca, New York, USA

**Keywords:** immune evasion, spillover, coronavirus, sarbecovirus, pangolin

## Abstract

Malayan pangolins are unusual hosts for several SARS-CoV-2-related coronaviruses, which have previously been known to only infect *Rhinolophus* bats. Much debate has hence surrounded their possible role as intermediate hosts in the emergence of SARS-CoV-2, but the virological phenotypes of most pangolin coronaviruses (pCoVs) remain unclear. Here, we comprehensively analyze all pCoVs to date identified from trafficked pangolins seized in the Guangdong province of China, which are remarkably similar to SARS-CoV-2 in the spike (S) protein. We explore the genetic diversity within these viruses and uncover how this diversity translates to different virological phenotypes. Strikingly, several Guangdong pCoVs harbor a lysine substitution at residue 519 of the S protein, which contributes to marked immune evasion by modulating the conformational state of the S protein. Furthermore, we highlight that a divergent immuno-evasive mutation at residue 519 of the S protein was acquired by SARS-CoV-2. These findings support that pangolin- and human-infecting coronaviruses likely represent independent spillover events from natural bat reservoirs, and that immuno-evasive mutations at residue 519 may be a common direction of viral evolution in coronaviruses that infect non-bat hosts.

**IMPORTANCE:**

Pangolins are frequently moved through illegal wildlife trade, creating opportunities for animal viruses to cross borders and encounter people. Guangdong pangolin coronaviruses are genetically close to SARS-CoV-2, particularly in spike, but their biological properties have been poorly defined. By analyzing all the available spike sequences of Guangdong pangolin coronaviruses and testing representative spikes in functional assays, we show that closely related pangolin coronaviruses can differ substantially in susceptibility to antibody neutralization. Notably, a single substitution at spike residue 519 can shift this phenotype by altering spike conformational dynamics, supporting the idea that residue 519 has been repeatedly targeted during adaptation outside bat reservoirs. These findings highlight spike residue 519 as a practical molecular marker to help flag immune-evasive, spillover-prone sarbecoviruses and to prioritize surveillance at wildlife-trade interfaces.

## INTRODUCTION

Severe acute respiratory syndrome coronavirus 2 (SARS-CoV-2) emerged at the end of 2019 and caused the coronavirus disease 2019 (COVID-19) pandemic. This global event drew much attention to coronavirus surveillance (1), which has yielded many viruses related to SARS-CoV-2. Independent of SARS-CoV-2, SARS-CoV also caused outbreaks mainly in East and Southeast Asian countries in 2002 (2). Both SARS-CoV and SARS-CoV-2 are classified into the family *Coronaviridae*, the genus *Betacoronavirus*, and the subgenus *Sarbecovirus* (3). Sarbecoviruses have been found to primarily infect bats belonging to the genus *Rhinolophus* (4–6), building on a long-standing consensus that *Rhinolophus* bats are a natural reservoir of sarbecoviruses (6). However, in recent years, a handful of sarbecoviruses have also been detected in smuggled Malayan pangolins (*Manis javanica*) in Southern China, specifically in the Guangdong (7–10) and Guangxi (7) provinces.

Malayan pangolins are distributed across much of Southeast Asia, sharing a common ecological niche and distribution with *Rhinolophus* bats (11). The proximity of Malayan pangolins to *Rhinolophus* bats in the wild places them in an environment where spillover of coronaviruses could occur. For instance, sarbecoviruses have been detected in *Rhinolophus* bats in Cambodia, geographically overlapping with pangolin populations (12). Furthermore, following the identification of pangolin coronaviruses (pCoVs in Southern China, SARS-CoV-2 neutralizing antibodies were identified in a pangolin in Southern Thailand (13) and in a pangolin carcass likely originating from Indonesia (14), suggesting that spillover events of coronaviruses into pangolin populations may occur naturally in Southeast Asia.

Malayan pangolins are among the most trafficked species globally (11). The illegal wildlife trade has allowed pangolins to act as a “vehicle” for various viruses (including coronaviruses) to cross country borders (15). As pangolins encounter wildlife smugglers, there is a risk that pCoVs may spill into human populations, causing future coronavirus disease emergence. Indeed, all pCoVs to date were identified using samples from seized pangolins of unknown country of origin in anti-smuggling operations (7–10).

The Guangdong pCoVs (GD pCoVs) were identified using samples from nine pangolins in March 2019 (7–10) and one pangolin in July 2019 (9), all sampled at the Guangdong Wildlife Rescue Center, China. On the other hand, the Guangxi pCoVs (GX pCoVs) were identified using samples originating from five pangolins seized by Guangxi Customs from August 2017 to January 2018 (7). The sequenced pCoVs were found to be highly similar to SARS-CoV-2 in the spike (S) protein, leading to debate surrounding whether pCoVs carried by smuggled pangolins played a role in the emergence of SARS-CoV-2 (9).

Previous studies into the pCoVs found that the virological phenotypes of pCoVs resemble those of SARS-CoV-2. For instance, similarly to SARS-CoV and SARS-CoV-2, both GD and GX pCoVs can use both pangolin and human angiotensin converting enzyme 2 (ACE2) as a functional receptor for infection (16). Additionally, certain GD pCoV strains are susceptible to neutralization by human anti-SARS-CoV-2 humoral immunity induced by SARS-CoV-2 infection and vaccination (17, 18). However, although several GD pCoV sequences identified from different pangolin individuals have been published (7–10), the virological phenotypes of most pCoVs remain unknown. In this study, we characterize the genotypic and phenotypic diversity of all GD pCoVs and analyze how these characteristics inform the evolution of sarbecoviruses in Malayan pangolins.

## RESULTS

### The GD pCoV RBD is closely related to the SARS-CoV-2 RBD

**Fig 1:**
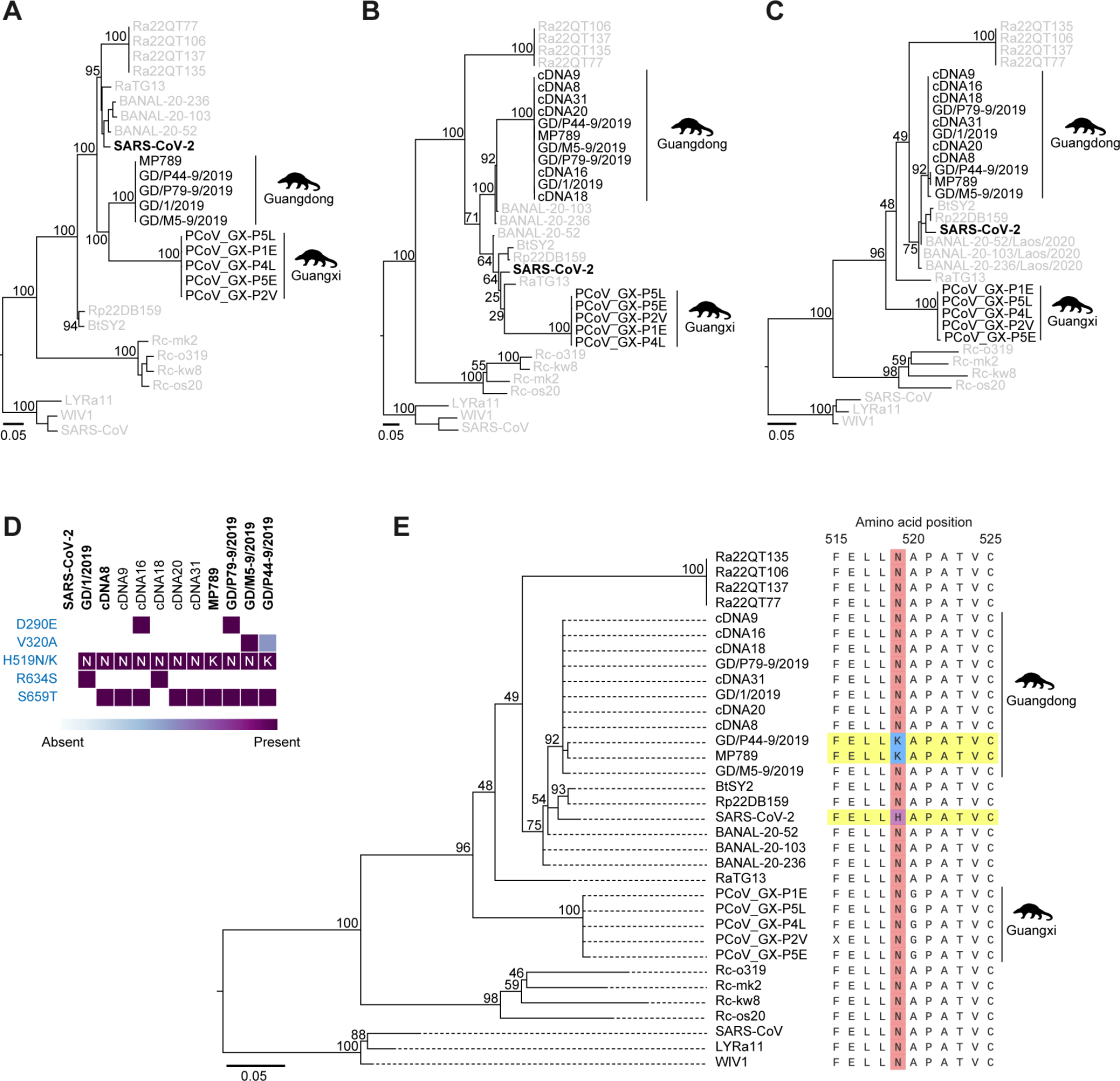
Phylogenetic relationship of GD pCoVs and differing sensitivity to human humoral immunity induced by SARS-CoV-2 vaccine. (**A–C**) Maximum likelihood (ML) trees of SARS-CoV-2-related pangolin coronaviruses, SARS-CoV-2 (strain Wuhan-Hu-1), and other sarbecoviruses. The trees based on the whole-genome sequences (**A**), S nucleotide sequences (**B**), and S RBD amino acid sequences (**C**) are shown. SARS-CoV-1 (strain Tor2) and two SARS-CoV-related coronaviruses (strains WIV1 and LYRa11) are included as an outgroup. SARS-CoV-2 is shown in bold. GD pCoVs and GX pCoVs are shown in black, and annotated based on previously published work (7–10). Other sarbecoviruses are shown in gray. Node support is shown above key branches. Scale bar indicates genetic distance (nucleotide/amino acid substitutions per site). (**D**) Amino acid substitutions in the S proteins of all 11 GD pCoVs detected to date. Substitutions and site positions are described in reference to SARS-CoV-2. The sequence of GD/P44-9/2019 has an ambiguous nucleotide resulting in a possible translation to alanine or valine at position 320. Since the alanine translation makes GD/P44-9/2019 identical to MP789, the valine translation at this position was adopted. The viruses in bold were used in this study. The substitutions in GD pCoVs and their surrounding amino acids are summarized in Fig. S1. (**E**) Amino acid residue positioned at 519 in Sarbecoviruses. Maximum likelihood trees of SARS-CoV-2-related pangolin coronaviruses, SARS-CoV-2 (strain Wuhan-Hu-1), and other sarbecoviruses based on the S RBD amino acid sequences. SARS-CoV-1 (strain Tor2) and two SARS-CoV-related coronaviruses (strains WIV1 and LYRa11) are included as an outgroup. Node support is shown above key branches. Scale bar indicates genetic distance (amino acid substitutions per site). The amino acid sequence alignment of the S protein from position 515 to position 525 is aligned to the tree. Sequences that do not harbor an asparagine at position 519 are highlighted in yellow. GD pCoVs and GX pCoVs are annotated based on previously published work (7–10).

We first set out to describe the phylogenetic relationship of pCoVs to other sarbecoviruses. A number of sarbecoviruses have been detected in *Rhinolophus* bats, but as of December 2025, only 16 pCoV whole genomes or S sequences, including 11 GD pCoVs and 5 GX pCoVs, have been published (7–10). To describe the phylogeny of pCoVs, we constructed three phylogenetic trees, which are based on the sequences of the whole genome (Fig. 1A), S protein (Fig. 1B), and S receptor-binding domain (RBD) (Fig. 1C). The strains and accession numbers are summarized in Table S1. All trees showed that all pCoVs are phylogenetically closer to SARS-CoV-2 (strain Wuhan-Hu-1) than SARS-CoV (strain Tor2) (Fig. 1A through C). Consistent with previous reports (7), pCoVs are phylogenetically separated into two clades corresponding to geography: GD pCoVs in Guangdong and GX pCoVs in Guangxi (Fig. 1A through C). Moreover, the molecular phylogenetic tree of the S RBD showed that all 11 GD pCoVs are more closely related to SARS-CoV-2 than GX pCoVs (Fig. 1C).

To compare the virological phenotypes of different GD pCoV strains, we compared all 11 of the publicly available GD pCoV S sequences. The sequences consist of three whole genomes (BetaCoV/pangolin/Guangdong/1/2019 [GD/1/2019] (10), MP789 (9), and GD/P79-9/2019 [8]), two partial genomes (GD/M5-9/2019 and GD/P44-9/2019) (8), and six S sequences (cDNA8, cDNA9, cDNA16, cDNA18, cDNA20, and cDNA31) (10). Compared to the SARS-CoV-2 S protein, GD pCoV S proteins display several substitutions among different strains, including D290E, V320A, H519N/K, R634S, and S659T (Fig. 1D). Two substitutions (V320A and H519N/K) are in the RBD (19), while one substitution (D290E) is in the N-terminal domain of S (19). While some GD pCoVs (e.g., GD/M5-9/2019 and GD/P44-9/2019) contain incomplete or ambiguous sequences, no other nonsynonymous mutations or indels were observed (Fig. S1).

Of the substitutions within GD pCoVs, the H519N/K substitution was notable as GD pCoVs harbored two different substitutions (asparagine and lysine) compared to histidine harbored by SARS-CoV-2 (Fig. 1E). Strikingly, all bat sarbecoviruses analyzed in this study harbored asparagine at position 519, suggesting that the common ancestor of both bat and pangolin sarbecoviruses harbored asparagine at position 519, while substitutions to lysine and histidine occurred independently of the later evolution of these viruses.

### GD pCoVs exhibit phenotypic diversity in susceptibility to humoral immunity induced by SARS-CoV-2 vaccination

We then constructed the plasmids expressing the S proteins of GD pCoVs as well as SARS-CoV-2, and prepared lentivirus-based pseudoviruses harboring these S proteins. It should be noted that the S amino acid sequences were identical for cDNA8, cDNA9, cDNA20, and cDNA31; for GD/P79-9/2019 and cDNA16; and for GD/1/2019 and cDNA18 (Fig. 1D). Therefore, cDNA8, GD/1/2019, and GD/P79-9/2019 were used as representatives of the strains with redundant S sequences.

**Fig 2:**
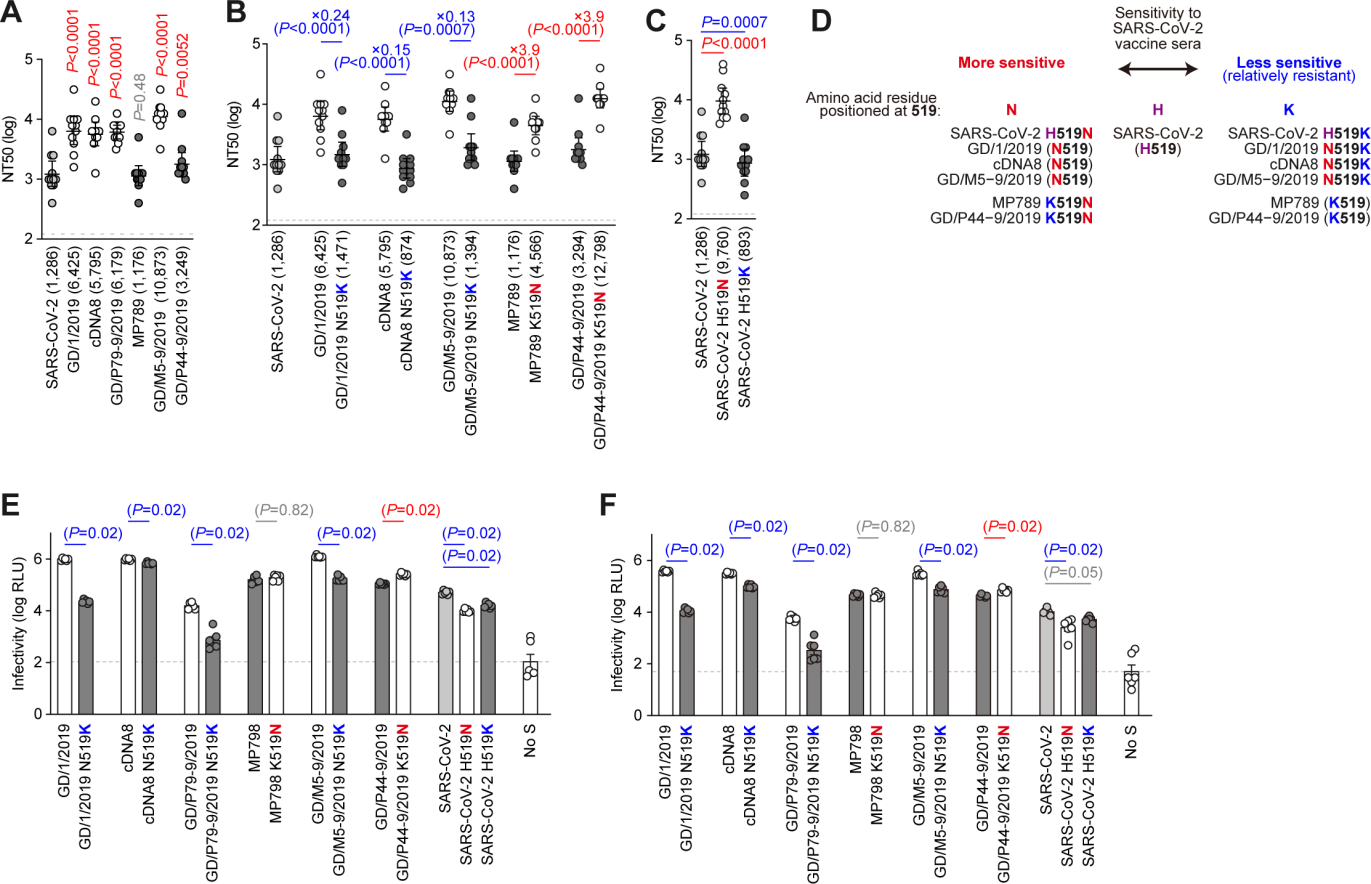
Effect of the amino acid substitution position at 519 on pseudovirus infectivity and sensitivity to SARS-CoV-2 vaccine sera. (**A**) Neutralization assay of SARS-CoV-2 and GD pCoVs. Neutralization assays were performed with pseudoviruses harboring the S proteins of GD pCoVs and SARS-CoV-2 against sera elicited by 3-dose Pfizer-BioNTech COVID-19 vaccination (*n* = 11). (**B**) Neutralization assay of GD pCoV 519 mutants. Neutralization assays were performed with pseudoviruses harboring the S proteins of GD pCoVs, SARS-CoV-2, and their derivatives against sera elicited by 3-dose Pfizer-BioNTech COVID-19 vaccination (*n* = 11). Due to total abrogated infectivity in the GD/P79-9/2019 N519K mutant, GD/P79-9/2019 was excluded from this neutralization assay. (**C**) Neutralization assay of SARS-CoV-2 519 mutants. Neutralization assays were performed with pseudoviruses harboring the S proteins of GD pCoVs, SARS-CoV-2, and their derivatives against sera elicited by 3-dose BNT162b2 Pfizer-BioNTech COVID-19 vaccination (*n* = 11). In panels A to C, assays for each serum sample were performed in triplicate to determine the 50% neutralization titer (NT50). Each point represents one NT50 value, and the geometric mean and 95% confidence intervals are shown. The number in parentheses indicates the geometric mean of NT50 values. The horizontal dashed line indicates the limit of detection (120-fold). Statistically significant differences versus SARS-CoV-2 (A and C) or parental strains (B) were determined by the two-sided Student’s t-test paired by serum samples, and *P* values are shown in the figure. Red and blue values indicate increased and decreased NT50s, respectively. The fold changes of NT50 versus parental are calculated as the average ratio of NT50s obtained from each pseudovirus. The fold changes versus parental are indicated with “X.” Information on the vaccine sera donors is summarized in Table S3. (**D**) Schematic summarizing the effect that the amino acid positioned at residue 519 (i.e., asparagine; N, histidine; H, or lysine; K) has on the sensitivity of pseudoviruses to vaccine sera. (**E and F**) Pseudovirus assay of GD pCoVs. HIV-1-based reporter viruses pseudotyped with the S proteins of GD pCoVs, SARS-CoV-2, and their derivatives were prepared. The pseudoviruses were inoculated into HOS-TMPRSS2 cells stably expressing human ACE2 (**E**) and HOS cells stably expressing human ACE2 (**F**). The mean and SD of the log-transformed pseudovirus infectivity are shown (representative of biological triplicate). The horizontal dashed line indicates the infectivity of the empty vector control. Assays were performed with six technical replicates. Statistically significant differences between variants and the parental strain were determined using the Wilcoxon signed-rank test, and *P* values are shown in the figure. Red and blue values indicate increased and decreased by the 519 substitutions, respectively. In panels A to C, E, and F, open circles/columns represent viruses that harbor an asparagine at position 519, while filled circles/columns represent viruses that harbor a lysine at position 519. SARS-CoV-2 is included as a control and is shaded in gray.

To compare the susceptibility of GD pCoVs to antiviral humoral immunity elicited by SARS-CoV-2 vaccination, we performed neutralization assays using sera from individuals who received three doses of the BNT162b2 Pfizer-BioNTech COVID-19 vaccine. As outlined in previous reports (17, 18), GD/1/2019 is susceptible to anti-SARS-CoV-2 humoral immunity induced by SARS-CoV-2 vaccination (Fig. 2A). Here, we showed that all GD pCoVs are sensitive to SARS-CoV-2 vaccine-induced humoral immunity (Fig. 2A). Notably, a one-way ANOVA revealed that the sensitivity of GD pCoVs to vaccine sera differs significantly between strains (*P* < 0.0001). While the 50% neutralization titer (NT50) of MP789 was comparable to that of SARS-CoV-2, vaccine sera exhibited significantly higher NT50s against GD/1/2019 (5.0-fold), cDNA8 (4.5-fold), GD/P79-9/2019 (4.8-fold), GD/M5-9/2019 (8.5-fold), and GD/P44-9/2019 (2.5-fold) compared to those against SARS-CoV-2 (Fig. 2A). Collectively, our results suggest that all GD pCoVs exhibited increased or comparable susceptibility to SARS-CoV-2 vaccine sera.

### Residue 519 is responsible for the increased sensitivity of some GD pCoVs to SARS-CoV-2 vaccine sera

To identify the amino acid residue(s) that determine the higher sensitivity of some GD pCoVs to SARS-CoV-2 vaccine sera, we again compared the amino acid sequences of GD pCoV S proteins. The GD pCoVs with higher sensitivity to vaccine sera (>4-fold; GD/1/2019, cDNA8, GD/P79-9/2019, and GD/M5-9/2019) all harbor asparagine (N) at residue 519 of S protein, while those with relatively comparable NT50 values to SARS-CoV-2 (MP789 and GD/P44-9/2019) harbor lysine (K) at this position (Fig. 1E and Fig. 2A, and Fig. S1). In contrast to all GD pCoVs, SARS-CoV-2 S harbors histidine (H) at residue 519. To investigate the impact of residue 519 on the immune susceptibility of GD pCoVs, we generated the following swapping substitutions at residue 519: The N519K derivatives for GD/1/2019, cDNA8, GD/P79-9/2019, and GD/M5-9/2019; the K519N derivatives for MP789 and GD/P44-9/2019; and the H519N and H519K derivatives for SARS-CoV-2.

We then assessed the influence of residue 519 on the sensitivity of GD pCoVs to SARS-CoV-2 vaccine sera. As shown in Fig. 2B, the N519K substitution in GD/1/2019, cDNA8, and GD/M5-9/2019 resulted in significantly lower NT50s exhibited by vaccine sera compared to parental S pseudoviruses. In sharp contrast, in the case of MP789 and GD/P44-9/2019, the K519N substitution resulted in significantly higher NT50s compared to parental S pseudoviruses (Fig. 2B). These results suggest that N519 confers higher sensitivity to vaccine sera, while K519 pseudoviruses exhibit lower immune sensitivity compared to GD pCoV pseudoviruses harboring parental S.

In the case of SARS-CoV-2 S, vaccine sera displayed significantly higher (7.6-fold) NT50s against the H519N derivative compared to parental SARS-CoV-2 (Fig. 2C). On the other hand, the H519K derivative exhibited significantly lower NT50s compared to parental SARS-CoV-2 (Fig. 2C). Altogether, these results suggest that the sensitivity to SARS-CoV-2 vaccine sera of both GD pCoV and SARS-CoV-2 S pseudoviruses can be modulated by residue 519 substitution of the S protein (Fig. 2D).

Next, we investigated how residue 519 affects the infectivity of GD pCoVs. We first performed Western blots of the S-expressing cells and confirmed successful expression of the S protein. There were differences observed in the expression of S protein between different GD pCoV pseudoviruses, but the S protein expression in parental SARS-CoV-2 and GD pCoV S-expressing cells, compared to the cells expressing their 519 variants, appeared to remain consistent (Fig. S2). We then performed infectivity assays using these pseudoviruses in cells expressing both human ACE2 and TMPRSS2, as well as in cells expressing human ACE2 alone. Consistent with previous reports (20), GD/1/2019 and MP789 were capable of infecting human ACE2-expressing cells. Here, we showed that all GD pCoVs can use human ACE2 as an infection receptor (Fig. 2E and F). The pseudovirus infectivity of MP789 was not significantly affected by the N/K swapping substitution at residue 519 in S. However, the N519K substitution conferred significantly decreased infectivity to GD/1/2019, cDNA8, GD/P79-9/2019, and GD/M5-9/2019, with nearly completely abrogated infectivity in GD/P79-9/2019 (Fig. 2E and F). In contrast, in the case of GD/P44-9/2019, the K519N substitution significantly increased infectivity compared to parental S pseudoviruses (Fig. 2E and F).

In the case of SARS-CoV-2, the H519N and H519K substitutions both significantly decreased pseudovirus infectivity (Fig. 2E and F). While other serine proteases besides TMPRSS2 could modulate the impact of 519 substitution (20), these results suggest that residue 519 modulates the pseudovirus infectivity of some GD pCoVs and SARS-CoV-2.

### Residue 519 substitution modulates the sensitivity to monoclonal antibodies recognizing its epitope

**Fig 3:**
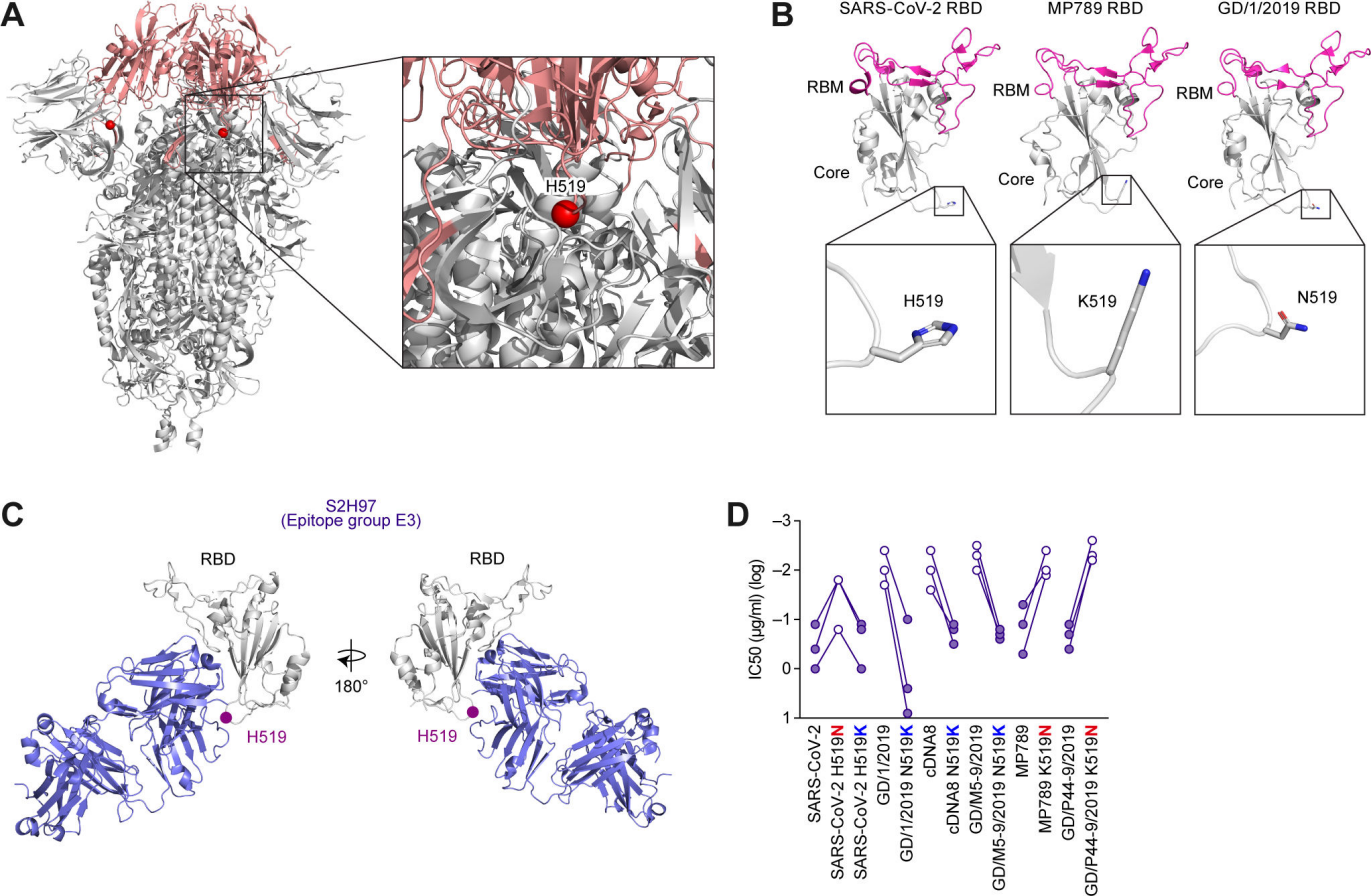
Effect of the amino acid substitution position at 519 on evasion from monoclonal antibodies targeting the epitope region including residue 519. (**A**) (Left) The cryo-EM structure of the SARS-CoV-2 S trimer. The complex structure is shown as a cartoon. (Right) The close-up view of the complex structure. The S RBD is highlighted in red and residue 519 is highlighted as a red sphere (PDB:6VXX). H519, histidine 519. (**B**) (Top) The crystal structure of SARS-CoV-2 S RBD (PDB:6M0J), cryo-EM structure of MP789 S RBD (PDB:7BBH), and cryo-EM structure of GD/1/2019 RBD (PDB:7CN8). (Bottom) Close-up views of the complex structures. Complex structures are shown as cartoons, and the residue positioned at 519 is shown as sticks. RBM, receptor-binding motif. (**C**) (Left) Crystal structure of SARS-CoV-2 S RBD and S2H97 (PDB:7M7W). The complex structure is shown as a cartoon, and residue 519 is shown as a red sphere. (Right) The structure rotated 180° on the y-axis. RBD, receptor-binding domain. (**D**) Neutralization assay using three monoclonal antibodies recognizing epitope group E3. Neutralization assays were performed with pseudoviruses harboring the S proteins of GD pCoVs, SARS-CoV-2, and their derivatives against three epitope group E3-targeting mAbs (BD55-3337, BD55-5415, and BD55-5583). Due to total abrogated infectivity in the GD/P79-9/2019 N519K mutant, GD/P79-9/2019 was excluded from this neutralization assay. Assays for each antibody were performed in triplicate to determine the 50% inhibitory concentration (IC50). Each point represents one log-transformed IC50 value, and IC50s of the same antibodies are connected by a solid line for derivatives of the same virus. Open circles represent viruses that harbor asparagine at position 519, while filled circles represent viruses that harbor lysine or histidine at position 519. Exact IC50 values are summarized in Table 1.

To address the question of how residue 519 modulates immune evasion, we aligned the S structures of SARS-CoV-2, MP789, and GD/1/2019. We utilized MP789 and GD/1/2019 as representatives of GD pCoVs harboring lysine and asparagine, respectively, at position 519. Residue 519 is located at the base of the RBD (residues 319–541 of the S protein) and is away from the ACE2 receptor-binding motif (RBM; residues 438–506 of the S protein), a region in the RBD that forms a binding interface with the ACE2 receptor (19) (Fig. 3A and B). Additionally, the position of residue 519 in the context of the RBD structure is comparable between SARS-CoV-2 (H519), MP789 (K519), and GD/1/2019 (N519) (Fig. 3B).

Next, we investigated the process by which residue 519 substitution impacts neutralization in SARS-CoV-2 and GD pCoVs. Since polyclonal vaccinated sera contain a mixture of multiple neutralizing antibodies, we utilized individual monoclonal antibodies (mAbs) against S RBD to characterize the effect of 519 substitution on different classes of neutralizing antibodies. In a deep mutational scanning screen of 1,538 mAbs against SARS-CoV-2 S pseudoviruses, Cao et al. (21) described 12 clusters of mAbs based on their escape mutations and dubbed them “epitope groups.” According to this definition, residue 519 is in the epitope group E3. To evaluate the effect of residue 519 substitution on the immunological phenotype of epitope group E3, we used three mAbs targeting this epitope. As shown in Fig. 3C, S2H97 (PDB:7M7W) (22), which is a classical mAb targeting epitope group E3, recognizes the structural region including residue 519 (21). As expected, the neutralization assay using these three mAbs showed that the pseudoviruses harboring N519 exhibited lower IC50s than those harboring K519 (Fig. 3D). Altogether, these results suggest that N519 confers higher sensitivity to the mAbs targeting the epitope group E3, while K519 pseudoviruses exhibit lower sensitivity to these mAbs.

### Residue 519 substitution indirectly affects neutralization activity by up-state RBD-binding monoclonal antibodies

We showed that the neutralization activity of antibodies recognizing epitope group E3 was dramatically improved by asparagine substitution at residue 519 (Fig. 3D). However, the neutralization activity of these mAbs has been reported to be relatively weak across a broad spectrum of SARS-CoV-2 variants (21). Moreover, since vaccinated sera contain a variety of neutralizing antibodies, E3-targeting mAbs alone may not fully explain the magnitude of immune sensitivity displayed by N519-harboring pseudoviruses. Therefore, we assumed that residue 519 substitution might also affect the neutralization activity of other mAbs in a different fashion.

**Fig 4:**
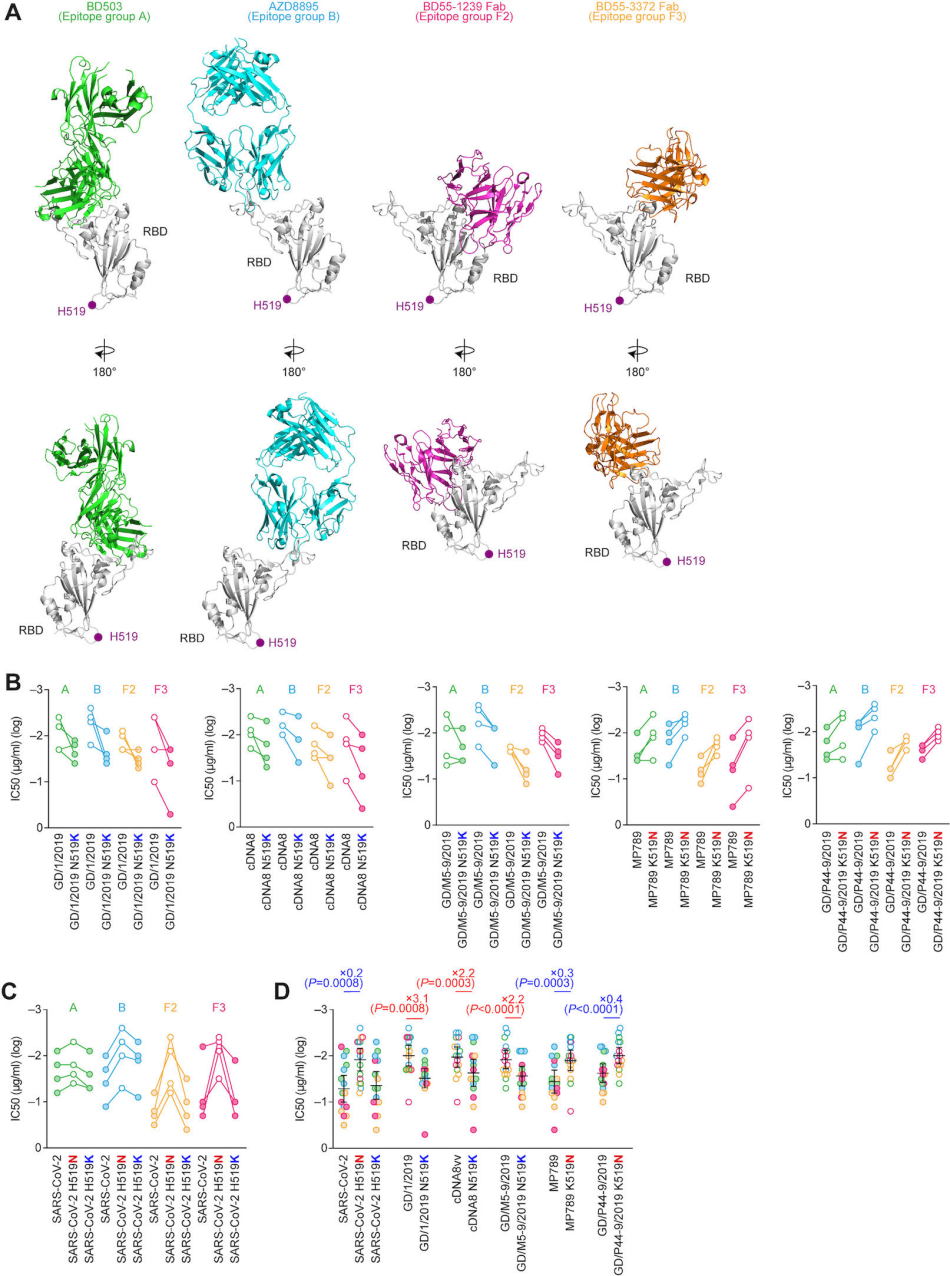
Effect of the amino acid substitution position at 519 on evasion from monoclonal antibodies potentially recognizing up-state RBD. (**A**) (Top) Structural model of SARS-CoV-2 S RBD in complex with BD-503 (PDB:7EJY), AZD8895 (PDB:7L7D), BD55-1239 (PDB:7WRL), and BD55-3372 (PDB:7WRO). The complex structure is shown as a cartoon, and histidine at residue 519 is shown as a purple sphere. (Bottom) The structures rotated 180° on the y axis. RBD, receptor-binding domain. (**B–D**) Neutralization assay using monoclonal antibodies recognizing epitope groups A, B, F2, and F3. Neutralization assays were performed with pseudoviruses harboring the S proteins of GD pCoVs, SARS-CoV-2, and their derivatives against a total of 16 monoclonal antibodies recognizing epitope groups A, B, F2, and F3. Assays for each antibody were performed in triplicate to determine the IC50. Due to total abrogated infectivity in the GD/P79-9/2019 N519K mutant, GD/P79-9/2019 was excluded from this neutralization assay. Each point represents one log-transformed IC50 value exhibited by the antibodies against GD pCoVs (**B**) and SARS-CoV-2 (**C**). Plots are separated by parental strain, and in each plot, the points are color-coded by epitope group. IC50s of the same antibodies are connected by a solid line for derivatives of the same virus. Open circles represent viruses that harbor asparagine at position 519, while filled circles represent viruses that harbor lysine or histidine at position 519. Exact IC50 values are summarized in Table 1. Statistical comparisons of the data shown in panels B and C are summarized in panel D. The geometric mean and 95% confidence intervals are shown. Statistically significant differences versus parental were determined by the two-sided Student’s t-test paired by antibody and *P* values are shown in the figure. Red and blue values indicate increased and decreased IC50s, respectively. The fold changes of IC50 versus parental are calculated as the average ratio of IC50 obtained from each pseudovirus. The fold changes versus parental are indicated with “X.” Exact IC50 values are summarized in Table 1.

The RBD of both GD pCoVs and SARS-CoV-2 can conformationally shift into an open (“up”) conformational state, with potent antibodies binding to sites that become more exposed in the up-state RBD conformation (23, 24). In fact, a previous study has suggested that residue 519 possibly modulates the up/down-state of the RBD (25). We therefore hypothesized that N519 may stabilize the up-state RBD conformation, consequently resulting in higher immune sensitivity to potent mAbs binding to sites exposed in the up-state RBD conformation. To investigate this hypothesis, we used mAbs targeting epitope groups A, B, F2, and F3 as described by Cao et al. (21). These mAbs bind at or near the RBM and compete with ACE2 by binding the up-state RBD conformation (Table 1) (21). Although residue 519 is not at the binding interface of these antibodies (Fig. 4A), we revealed that most of the up-state RBD-targeting mAbs exhibited higher IC50 values against the N519K derivatives of GD/1/2019 (13 out of 16), cDNA8 (15 out of 16), and GD/M5-9/2019 (14 out of 16) compared to parental S pseudoviruses (Fig. 4B and Table 1). In contrast, these mAbs exhibited lower IC50 values against the K519N derivatives of MP789 (15 out of 16) and GD/P44-9/2019 (15 out of 16) compared to parental S pseudoviruses (Fig. 4B and Table 1). In the case of SARS-CoV-2, H519N led to all 16 of the up-state RBD-targeting mAbs exhibiting lower IC50 values (Fig. 4C and Table 1). Collectively, our results suggest that N519 indirectly affects the conformation of RBD, thereby enhancing neutralization by up-state RBD-binding neutralizing antibodies in GD pCoVs and SARS-CoV-2 (Fig. 4D).

**TABLE 1:** GD pCoV and SARS-CoV-2 neutralization by monoclonal antibodies

Antibody ID	Epitope group^*a*^	ACE2 competition^*a*^	IC50 (μg/mL)												
			GD/1/2019	GD/1/2019 N519K	cDNA8	cDNA8 N519K	MP789	MP789 K519N	GD/M5-9/2019	GD/M5-9/2019 N519K	GD/P44-9/2019	GD/P44-9/2019 K519N	SARS-CoV-2	SARS-CoV-2 H519N	SARS-CoV-2 H519K
BD55-3337	E3	0.03	0.010	2.457	0.010	0.308	0.504	0.012	0.005	0.224	0.362	0.005	1.016	0.150	1.073
BD55-5415	E3	0.01	0.020	8.726	0.028	0.172	0.048	0.011	0.009	0.212	0.218	0.007	0.115	0.017	0.122
BD55-5583	E3	0.01	0.004	0.097	0.004	0.123	0.133	0.004	0.003	0.159	0.113	0.002	0.444	0.017	0.146
BD30-503	A	0.86	0.006	0.011	0.010	0.017	0.031	0.013	0.008	0.009	0.017	0.005	0.015	0.015	0.028
BD30-504	A	0.62	0.022	0.016	0.009	0.053	0.041	0.010	0.034	0.036	0.033	0.020	0.032	0.024	0.056
BD55-300	A	0.92	0.004	0.026	0.004	0.005	0.011	0.004	0.004	0.019	0.009	0.004	0.008	0.006	0.008
BD30-605	A	0.57	0.019	0.045	0.020	0.033	0.043	0.038	0.048	0.041	0.045	0.044	0.063	0.045	0.051
BD55-5463	B	0.74	0.005	0.007	0.006	0.013	0.014	0.006	0.006	0.007	0.007	0.005	0.131	0.051	0.077
BD55-203	B	0.8	0.014	0.023	0.011	0.041	0.050	0.011	0.018	0.047	0.053	0.010	0.038	0.011	0.011
BD55-5966	B	0.89	0.002	0.033	0.003	0.004	0.006	0.005	0.003	0.007	0.006	0.003	0.019	0.005	0.009
BD55-6382	B	0.82	0.004	0.040	0.003	0.004	0.011	0.004	0.003	0.007	0.006	0.003	0.011	0.003	0.005
BD55-1239	F2	0.82	0.022	0.018	0.016	0.034	0.122	0.020	0.028	0.119	0.109	0.027	0.217	0.037	0.181
BD55-3500	F2	0.91	0.022	0.034	0.028	0.034	0.064	0.034	0.024	0.066	0.067	0.014	0.343	0.064	0.393
BD55-5380	F2	0.81	0.009	0.044	0.006	0.009	0.031	0.016	0.022	0.026	0.022	0.015	0.154	0.004	0.093
BD55-5189	F2	0.77	0.008	0.047	0.028	0.130	0.076	0.013	0.020	0.082	0.069	0.016	0.069	0.007	0.035
BD55-4637	F3	0.81	0.019	0.019	0.013	0.077	0.062	0.013	0.015	0.077	0.039	0.015	0.103	0.031	0.090
BD55-3372	F3	0.88	0.004	0.020	0.004	0.009	0.012	0.005	0.009	0.018	0.018	0.009	0.006	0.006	0.012
BD30-449	F3	0.75	0.109	0.470	0.108	0.410	0.426	0.144	0.013	0.034	0.025	0.013	0.197	0.008	0.183
BD55-5472	F3	0.73	0.004	0.040	0.015	0.021	0.053	0.011	0.010	0.026	0.019	0.010	0.125	0.004	0.103

^*a*^ Epitope group and ACE2 competition levels were determined by Cao et al. (21). Their findings for the mAbs utilized in this study are reported here.

### Residue 519 substitution modulates RBD conformational dynamics

To quantify how residue 519 modulates the RBD up/down equilibrium, we performed ΔΔG scanning with Rosetta (26) on all-down (closed) and one-up (open) S conformations using SARS-CoV-2, MP789 as a representative of K519 pCoVs, and GX-P2V as a representative of N519 pCoVs. For each conformation, we computed the mutation-induced stability change relative to the wild type in each state and then evaluated a preference metric (ΔΔG_closed_ − ΔΔG_open_). By definition, negative values indicate that the mutation stabilizes the closed state more than the open state, whereas positive values indicate a relative stabilization of the open state.

For ancestral SARS-CoV-2 (H519), substituting lysine at 519 strongly favored the closed state: ΔΔG_closed_ = −2.2 REU and ΔΔG_open_ = 10.5 REU, yielding ΔΔG_closed_ − ΔΔG_open_ = −12.7 REU. In contrast, H519N produced positive ΔΔG values in both states (7.5 and 6.7 REU), with a near-neutral difference of +0.8 REU. Together, these data indicate that lysine at 519 stabilizes the all-down conformation in the ancestral strain, whereas asparagine is destabilizing with little state preferences (Table 2).

**TABLE 2:** Computational assessment of a mutation at 519 in the S protein of ancestral SARS-CoV-2, GX-P2V, and MP789

Strain and residue	REU		
	∆∆G_closed_	∆∆G_opened_	∆∆G_closed_ – ∆∆G_opened_
Ancestral SARS-CoV-2^*a*^ (harboring **H519**)			
H519K	−2.2	10.5	−12.7
H519N	7.5	6.7	0.8
GX-P2V^*b*^ (harboring **N519**)			
N519K	-7	−3.5	−3.5
MP789^*c*^ (harboring **K519**)			
K519N	7	−3.5	10.5

^*a*^ Data for ∆∆G_closed_ are from PDB identifier 6VXX; data for ∆∆G_opened_ are from PDB identifier 6XM4.

^*b*^ Data for ∆∆G_closed_ are from PDB identifier 7CN8; data for ∆∆G_opened_ are from our structural model.

^*c*^ Data for ∆∆G_closed_ are from PDB identifier 7BBH; data for ∆∆G_opened_ are from our structural model.

In GX-P2V (N519), N519K stabilized both conformations but favored the closed state (−7.0 vs −3.5 REU; difference −3.5 REU). In MP789 (K519), K519N destabilized the closed state while stabilizing the open state (7.0 vs −3.5 REU; difference +10.5 REU), predicting a pronounced shift toward the one-up conformation. These results across lineages are consistent with the analysis of ancestral SARS-CoV-2 and support the view that introducing a positive charge at 519 biases the S toward the all-down conformation (Table 2).

### Residue 519 substitution impacts ACE2 binding

**Fig 5:**
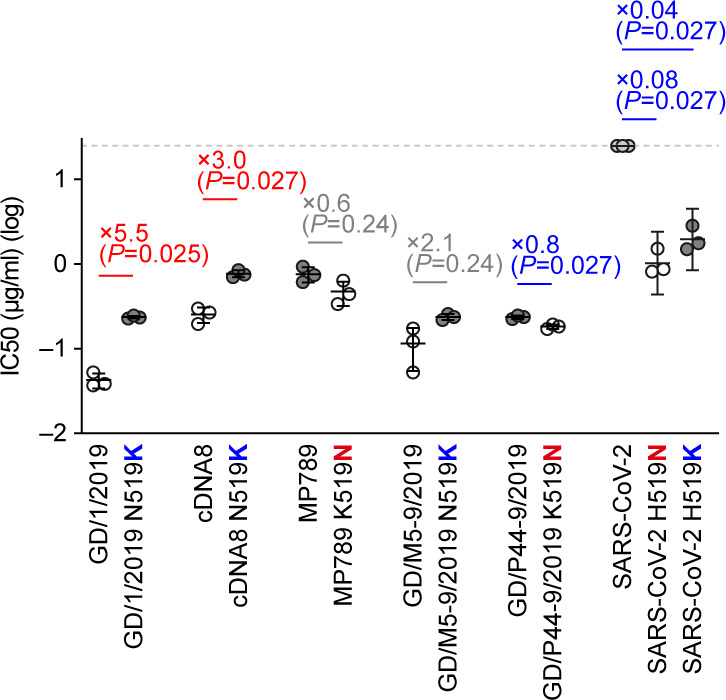
Effect of the amino acid substitution position at 519 on ACE2 binding and infectivity in human ACE2-expressing cells. Neutralization assay using soluble ACE2. Three biological replicates of the neutralization assay were performed with pseudoviruses harboring the S proteins of GD pCoVs, SARS-CoV-2, and their derivatives against soluble ACE2. Each biological replicate was performed in triplicate to determine the IC50. Due to total abrogated infectivity in the GD/P79-9/2019 N519K mutant, GD/P79-9/2019 was excluded from this neutralization assay. Each point represents one IC50 value exhibited by soluble ACE2 against GD pCoVs and SARS-CoV-2. Plots are separated by parental strain. Open circles/columns represent viruses that harbor an asparagine at position 519, while filled circles/columns represent viruses that harbor a lysine at position 519. SARS-CoV-2 is included as a control and is shaded in gray. The horizontal dashed line indicates the upper limit of detection (25 µg/mL). Statistically significant differences between variants and the parental strain were determined using Welch’s t-test on the log IC50 values, and *P* values are shown in the figure. Red and blue values indicate increased and decreased by the 519 substitutions, respectively. The fold changes of IC50 versus parental are calculated as the average ratio of IC50 obtained from each pseudovirus. The fold changes versus parental are indicated with “X.”

Conformational changes favoring the up-state RBD are known to increase access for the binding of both up-state RBD-targeting mAbs and ACE2 (25). To address whether ACE2 binding is also affected by N519 in GD pCoVs, we performed neutralization assays using soluble ACE2. As outlined in previous reports (27), higher neutralization by soluble ACE2 is correlated with a greater binding affinity for cell-bound ACE2, allowing sensitivity to neutralization by soluble ACE2 to be used as a proxy for ACE2 binding affinity. Although position 519 is not at the receptor binding motif of ACE2, we observed that 519 substitutions alter sensitivity to soluble ACE2 neutralization. Across three biological replicates, the N519 GD pCoVs GD/1/2019 and cDNA8 were neutralized by soluble ACE2 significantly more effectively than their K519 variants, while the K519 GD pCoV GD/P44-9/2019 was neutralized significantly less effectively than its N519 variant (Fig. 5). These results suggest that N519 substitution improves whole S ACE2 binding in GD pCoVs.

In SARS-CoV-2, the H519N variant was neutralized by soluble ACE2 significantly more effectively than parental SARS-CoV-2 S pseudoviruses (Fig. 5), suggesting that the H519N substitution improves whole S ACE2 binding in SARS-CoV-2. On the other hand, the SARS-CoV-2 S H519K was also more sensitive to soluble ACE2 (Fig. 5). These results suggest that the impact of residue 519 on SARS-CoV-2 is not completely similar to that on GD pCoV S.

## DISCUSSION

Previous studies involving pCoVs have utilized either GD/1/2019 (18, 24) or MP789 (28) as a representative. In this study, we elucidate the previously unknown phenotypic diversity in all GD pCoVs known to date. We showed that the neutralization sensitivity of GD pCoVs differs between strains. Furthermore, we experimentally demonstrated that these differences are determined by the amino acid residue positioned at 519. Importantly, the impact of residue 519 is not restricted to GD pCoVs but is also observed in SARS-CoV-2.

The evolutionary implications of residue 519 substitution are striking in the context of the broader genetic landscape of sarbecoviruses. Asparagine at position 519 (N519) is remarkably conserved in most sarbecoviruses, and the S proteins of all bat sarbecoviruses analyzed in this study harbored N519 (Fig. 1E). As an exception to this trend, certain GD pCoVs harbor K519, and SARS-CoV-2 harbors H519. These observations suggest that SARS-CoV-2-related coronaviruses seem to prefer substituting residue 519 once out of their bat hosts. Because we find that residue 519 possibly modulates the up/down-state of the RBD (Table 2) (25), conformational switching by residue 519 substitution may be critical for sarbecoviruses to spill over into non-bat hosts, including pangolins and humans. H519 has remained remarkably conserved in SARS-CoV-2, being present in SARS-CoV-2 Wuhan-Hu-1 all the way up to presently dominant variants at the time of writing, including the SARS-CoV-2 JN.1 subvariants XFG and NB.1.8.1 (29). Since we find that H519N both increases immune sensitivity and lowers infectivity, it is no surprise that this reverting substitution has not been widely observed in the ongoing evolution of SARS-CoV-2.

Since we find that K519 drives immune evasion, the evasion of antiviral humoral immunity in pangolins may have been a key factor driving the acquisition of K519 in some GD pCoVs. Supporting this idea, Wacharapluesadee et al. (13) have detected antibodies that cross-react with SARS-CoV-2 in pangolin serum in Southern Thailand. Since the serological and/or virological prevalence of pCoVs in wild pangolins in Southeast Asia remains unclear, it is unknown whether there is pre-existing anti-pCoV immunity in wild pangolins. However, it might be possible to assume that the K519 substitution was acquired to evade pre-existing anti-pCoV humoral immunity in wild pangolins. Since early SARS-CoV-2 sequences all harbor H519 (30), this also raises the critical question of whether the substitution from asparagine to histidine occurred in a putative intermediate host or shortly after the emergence of SARS-CoV-2 in humans.

In addition to differences in immune susceptibility, pseudovirus infectivity was also different between the GD pCoV strains: the N519K substitution significantly decreased pseudovirus infectivity in some N519 viruses (GD/1/2019, cDNA8, GD/P79-9/2019, GD/M5-9/2019) and vice versa (K519N; GD/P44-9/2019) but did not significantly affect the pseudovirus infectivity of MP789 (Fig. 2E and F) both in the presence and absence of TMPRSS2. Therefore, the differences in pseudovirus infectivity brought by residue 519 in GD pCoVs may be determined not only by residue 519 but also by epistatic interactions of residue 519 with other residues in the S protein, including those located outside the RBD. Surprisingly, in the case of SARS-CoV-2, the H519N and H519K substitutions both significantly decreased pseudovirus infectivity (Fig. 2E and F), despite H519N displaying higher whole S ACE2 binding. These results are consistent with a previous report by Cereghino et al. (31), where they report that the H519N SARS-CoV-2 S RBD displays lower ACE2 binding than the parental RBD. In combination with their results, this suggests that while H519N may drive the S RBD into the up-RBD position, it may also allosterically modify the S RBD, resulting in lower engagement of ACE2 by the S RBD. Furthermore, the lowered infectivity of H519N SARS-CoV-2 S pseudoviruses despite more efficient whole S ACE2 binding suggests that substitutions at residue 519 may also disrupt other aspects of the cell entry process besides ACE2 binding, consistent with a previous report suggesting that pseudovirus whole S ACE2 binding and cell entry are distinct phenotypes (32). ΔΔG scanning with Rosetta also predicted H519N to be destabilizing to the S trimer overall, which may contribute to the complex effects of this substitution in SARS-CoV-2.

Limitations of this study include that we did not describe how the H519N substitution in SARS-CoV-2 could both stabilize the up-RBD conformation and reduce cell entry simultaneously. Further studies into how cell fusion is affected by residue 519 substitution are needed to characterize the full effect of the 519 substitution. In GD pCoVs, we also found differences in the S protein expression amounts between different GD pCoV strains, which presented a confounding factor in performing comparisons between different GD pCoV strains. Another limitation of this study is that we assessed S protein expression in producer cell lysates but did not directly evaluate S incorporation into pseudovirus particles. Therefore, we cannot exclude the possibility that differences in S incorporation may contribute to the observed differences in pseudovirus infectivity.

In conclusion, our study explored a heretofore unknown phenotypic diversity within the pCoVs detected in the Guangdong province of China. We demonstrated that substitutions within GD pCoVs contribute to differences in the infectivity and immune sensitivity of these viruses. Notably, we identified a substitution from asparagine to lysine at position 519 as a key driver of lowered immune sensitivity. This suggests that lysine substitution at position 519 is a potent immune escape mutation harbored by GD pCoVs, including MP789 and GD/P44-9/2019, supporting that further surveillance of pCoVs, especially those harboring K519, should be a key component of measures to anticipate and mitigate future outbreaks. Nevertheless, it should be noted that SARS-CoV-2 vaccine sera are still capable of satisfactorily neutralizing all GD pCoVs. The intricacies of the immunity of bats, pangolins, putative intermediate hosts, and humans, which make it evolutionarily advantageous for residue 519 substitutions to occur in non-bat hosts, should also be further explored. The use of pangolin sera would be instrumental to furthering this work, but since Malayan pangolins are a critically endangered species, great caution must be employed in the acquisition and use of such samples.

## MATERIALS AND METHODS

### Nucleotide sequence data collection

The nucleotide sequences of sarbecoviruses with *Manis javanica* as their host were retrieved from NCBI virus (download date, 11 November 2024). In addition, SARS-CoV-2 (Wuhan-Hu-1) and relevant sarbecovirus sequences were collected from NCBI GenBank (download date, 11 November 2024). GD/1/2019 (10) was accessed from GISAID on 6 February 2025 (GISAID ID: EPI_ISL_410721). Information on the sarbecovirus sequences is summarized in Table S1.

### Phylogenetic analysis

To evaluate the phylogenetic relationships between all sarbecoviruses, we aligned the complete genomes using the default option of MAFFT v7.526 (33). The aligned sequence was cleaned using an in-house Python script to replace any characters not in “ATGCN-” with “N.” Next, we inferred an ML tree based on this alignment using IQ-TREE2 v2.2.6 (34) under a GTR+F+I+R4 substitution model (Fig. 1A). Node support was assessed with 1,000 Ultrafast bootstrap replicates (35). Next, we extracted the S-encoding part of the genomes (using the SARS-CoV-2 Wuhan-Hu-1 strain S protein as a reference) and used the method above to construct an S tree based on their nucleotide sequences (Fig. 1B). We then manually extracted the RBD sequences from the S gene using the SARS-CoV-2 RBD as a reference (i.e., amino positions 319–541 in the SARS-CoV-2 Wuhan-Hu-1 strain S protein) and constructed an amino acid alignment using MAFFT v7.526 (33). This alignment was used to create an ML tree using IQ-TREE2 v2.2.6 (34) under the WAG + R2 model selected by ModelFinder (36) with 1,000 bootstrap iterations.

### Amino acid sequence alignment

To assess the similarity between all GD pCoVs, we utilized the sequences of SARS-CoV-2 Wuhan-Hu-1, GD/1/2019, cDNA8, cDNA9, cDNA16, cDNA18, cDNA20, cDNA31, MP789, GD/P79-9/2019, GD/M5-9/2019, and GD/P44-9/2019. Sequence information is summarized in Table S1. We extracted the S-encoding part of the genomes (using the SARS-CoV-2 Wuhan-Hu-1 strain S protein as a reference) for the GD pCoV sequences above and constructed an amino acid sequence alignment using MAFFT v7.526 (33) with the GTR+F+I+R4 substitution model, using 1,000 maximum iterative refinements. Then, the substitutions within the GD pCoV sequences were manually confirmed in reference to the SARS-CoV-2 Wuhan-Hu-1 amino acid sequence (Fig. S1).

### Plasmid construction

Plasmids expressing the codon-optimized S proteins of GD pCoVs were synthesized by a gene synthesis service (Fasmac). The plasmid expressing the codon-optimized SARS-CoV-2 S protein (strain Wuhan-Hu-1; GenBank accession number: NC_045512.2) (30) was kindly provided by Dr. Kenzo Tokunaga. Plasmids expressing the residue 519 swapping substitutions of the S proteins of SARS-CoV-2 and GD pCoVs were generated by site-directed overlap extension PCR using the templates and primers listed in Table S2. The resulting mutagenized PCR fragments were cloned into the KpnI/NotI site of the pCAGGS vector (37) using the In-Fusion HD Cloning Kit (Takara, Cat# Z9650N). Nucleotide sequences were determined by DNA sequencing services (Eurofins), and the sequence data were analyzed by SnapGene v8.0.1 (SnapGene software).

### Cell culture

Lenti-X 293T cells (a human embryonic kidney cell line; ATCC, Takara, Cat# 632180) and HOS-ACE2/TMPRSS2 cells (kindly provided by Dr. Kenzo Tokunaga), a derivative of HOS cells (a human osteosarcoma cell line; ATCC CRL-1543) stably expressing human ACE2 and TMPRSS2 (38, 39) were maintained in Dulbecco’s modified Eagle’s medium (high glucose) (Sigma-Aldrich, Cat# 6429-500ML) containing 10% fetal bovine serum (Sigma-Aldrich Cat# 172012-500ML) and 1% penicillin-streptomycin (Sigma-Aldrich, Cat# P4333-100ML).

### Pseudovirus assay

Pseudoviruses were prepared as previously described (40–43). Briefly, lentivirus (HIV-1)-based luciferase-expressing reporter viruses were pseudotyped with the S proteins of GD pCoVs or SARS-CoV-2 and their derivatives. LentiX-293T cells (500,000 cells) were cotransfected with 0.8 μg psPAX2-IN/HiBiT (39), 0.8 μg pWPI-Luc2 (44), and 0.4 μg plasmids expressing parental S or its derivatives using TransIT-293 (Takara, Cat# MIR2704) according to the manufacturer’s protocol. Two days post-transfection, the culture supernatants were harvested, and the pseudoviruses were stored at –80°C until use. For pseudovirus infection, the amount of input virus was normalized to the HiBiT value measured by the NanoGlo HiBiT lytic detection system (Promega, Cat# N3040) as previously described (44). In this system, HiBiT peptide is produced with HIV-1 integrase and forms NanoLuc luciferase with LgBiT, which is supplemented with substrates. In each pseudovirus particle, the detected HiBiT value is correlated with the amount of the pseudovirus capsid protein, HIV-1 p24 protein (44). Based on the HiBiT value measured, we calculated the amount of HIV-1 p24 capsid protein according to the previous paper (44). In the pseudovirus infection assays, pseudoviruses were normalized to 4 ng of HIV-1 p24 capsid protein. At 2 days post-infection, the infected cells were lysed with a BrightGlo luciferase assay system (Promega, Cat# E2620), and the luminescent signal produced by firefly luciferase reaction was measured using a GloMax Explorer Multimode Microplate Reader (Promega).

### Human serum collection

Vaccine sera were collected from individuals who had received three doses of the Pfizer-BioNTech COVID-19 vaccine (time interval between the last vaccination and sampling: 16–33 days; *n* = 11; average age: 39.0 years, range: 29–53 years, 36.4% male). Sera were inactivated at 56°C for 30 minutes and stored at –80°C until use. The details of the vaccine sera are summarized in Table S3.

### Antibody isolation and recombinant production

The 19 monoclonal antibodies used in this study (listed in Table 1) were kindly provided by Dr. Yunlong Cao. These antibodies were generated as previously described by Cao et al. (21).

### Neutralization assay

Pseudovirus neutralization assays against vaccine sera, mAbs, and soluble ACE2 were performed as previously described (45) Briefly, the GD pCoV and SARS-CoV-2 S pseudoviruses (counting ~ 100,000 relative light units) were incubated with serially diluted (120-fold to 87,480-fold dilution at the final concentration) heat-inactivated sera; serially diluted (0.008-fold to 125-fold the IC50 reported by Cao et al. 21() at the final concentration) mAbs; or 0.0016–25 µg/mL hACE2-Fc dimer (10108-H02H, Sino Biological) at 37°C for 1 hour. Pseudoviruses without serum/mAbs/ACE2 were included as controls. Then, a 20 µL mixture of pseudovirus and serum/mAbs/ACE2 was inoculated into HOS-TMPRSS2 cells stably expressing human ACE2 (10,000 cells/100 µL) in a 96-well white plate. Two days post-infection, the infected cells were lysed with a Bright-Glo luciferase assay system (Promega, Cat# E2620), and the luminescent signal was measured using a GloMax explorer multimode microplate reader 3500 (Promega). The assay of each serum sample was performed in triplicate, and the 50% neutralization titer (NT50) or the IC50 was calculated using Prism 10 software v10.3.1 (GraphPad Software).

### Protein structure model

In Fig. 3A, the cryo-EM structure of the S trimer of SARS-CoV-2 was used (PDB: 6VXX) (46). In Fig. 3B, the crystal co-structure of SARS-CoV-2 S (PDB:6M0J) (19), cryo-EM structure of MP789 S (PDB: 7BBH) (28), and cryo-EM structure of GD/1/2019 S (PDB: 7DDO) (16) were used. In Fig. 3C, the structure of S2H97 (PDB: 7M7W) (22) was used. In Fig. 4A, the structure of BD-503 (PDB: 7EJY) (45), structure of AZD8895 (PDB: 7L7D) (47), structure of BD55-1239 Fab (PDB: 7WRL) (21), and the structure of BD55-3372 Fab (PDB: 7WRO) (22) were used. All protein structural analyses were performed using the PyMOL molecular graphics system v3.0.0 (Schrödinger).

### ΔΔG scanning in closed and open states

For the ancestral SARS-CoV-2, we used the all-down (closed) S trimer (6VXX) and the one-up (open) S (6XM4). For GX-P2V (N519) and MP789 (K519), closed-state models were taken from the Protein Data Bank (7CN8 and 7BBH, respectively). Open-state models for these two strains were generated by mapping all the sequences of each strain onto the backbone of 6XM4, followed by side-chain repacking with Rosetta (26). All comparisons within a strain were performed on sequence-matched closed and open models. All models were first subjected to Cartesian relaxation with coordinate constraints for backbone atoms (48) while preserving the target conformation (closed vs one-up). Mutation scans at position 519 were carried out with the Rosetta Cartesian ΔΔG protocol (49) on each conformational state. For the ancestral SARS-CoV-2 (H519), H519K and H519N were evaluated. For GX-P2V (N519), N519K was evaluated. For MP789 (K519), K519N was evaluated. For each state, ΔΔG was defined as the difference in Rosetta energy units (REU) between the mutant and the corresponding wild type on the same structural model. The state-preference metric was computed as follows:


$$\Delta \Delta G_{closed}-\Delta \Delta G_{open}$$

with negative values interpreted as favoring all-down (closed) and positive values as favoring one-up (open).

### Western blot

Western blot was performed as previously described (50). Transfected LentiX-293T cells (see “Pseudovirus assay,” above) were detached and washed twice with PBS. The harvested cells were then washed and lysed in RIPA buffer (50 mM Tris-HCl buffer [pH 7.6], 150 mM NaCl, 1% Nonidet P-40, 0.5% sodium deoxycholate, 0.1% SDS) containing a protease inhibitor cocktail (Nacalai Tesque, Cat# 03969-21)]. The lysates were diluted with 2× sample buffer (100 mM Tris-HCl [pH 6.8], 4% SDS, 12% β-mercaptoethanol, 20% glycerol, 0.05% bromophenol blue). Samples were boiled for 10 minutes. Then, 10 μL samples were subjected to Western blot. For protein detection, the following antibodies were used: mouse anti-alpha-tubulin (TUBA) monoclonal antibody (clone DM1A, Sigma-Aldrich, Cat# T9026, 1:10,000), mouse anti-SARS-CoV-2 S monoclonal antibody (clone 1A9, GeneTex, Cat# GTX632604, 1:10,000), and HRP-conjugated horse anti-mouse IgG antibody (Cell Signaling, Cat# 7076S, 1:2,000). Chemiluminescence was detected using SuperSignal West Femto Maximum Sensitivity Substrate (Thermo Fisher Scientific, Cat# 34095) or Western Lightning Plus-ECL (PerkinElmer, Cat# NEL104001EA) according to the manufacturer’s instructions. Bands were visualized using the ChemiDoc Touch Imaging System (Bio-Rad).

### Statistical analysis

Statistical significance in the neutralization assays was tested using the Wilcoxon signed-rank test on log-transformed NT50 and IC50 values. NT50 and IC50 values from the same serum samples and mAbs, respectively, were paired. Statistical significance in the pseudovirus infectivity assay was tested using the Wilcoxon signed-rank test on log-transformed luminescence values. Statistical significance in the ACE2 binding assay was tested using Welch’s t-test on log-transformed IC50 values. All *P* values were adjusted to account for multiple comparisons using the Holm-Šídák method. The tests above were performed using Prism 10 software v10.3.1 (GraphPad Software). *P* < 0.05 was considered significant.

## Data Availability

All databases/datasets used in this study are available from the GenBank database (https://www.ncbi.nlm.nih.gov/nuccore/?term=txid2509511[Organism:exp]) and the GISAID database (https://www.gisaid.org; EPI_ISL_410721). All raw files used in this study are available on the GitHub repository (https://github.com/TheSatoLab/pCoV_519). Any additional information required to reanalyze the data reported in this work is available from the lead contact upon request.

## SUPPLEMENTAL MATERIAL

Supplemental figures jvi.00352-26-s0001.pdf Fig. S1 and S2.

Table S1 jvi.00352-26-s0002.docx Sequence data and accession numbers used in this study.

Table S2 jvi.00352-26-s0003.docx Primers used in this study.

Table S3 jvi.00352-26-s0004.docx Human three-dose Pfizer-BioNTech vaccine sera used in this study.

ASM does not own the copyrights to Supplemental Material that may be linked to, or accessed through, an article. The authors have granted ASM a non-exclusive, world-wide license to publish the Supplemental Material files. Please contact the corresponding author directly for reuse.

## References

[1] Crits-Christoph A, Levy JI, Pekar JE, Goldstein SA, Singh R, Hensel Z, Gangavarapu K, Rogers MB, Moshiri N, Garry RF, Holmes EC, Koopmans MPG, Lemey P, Peacock TP, Popescu S, Rambaut A, Robertson DL, Suchard MA, Wertheim JO, Rasmussen AL, Andersen KG, Worobey M, Débarre F. 2024. Genetic tracing of market wildlife and viruses at the epicenter of the COVID-19 pandemic. Cell 187:5468–5482. doi:10.1016/j.cell.2024.08.010 39303692 PMC11427129

[2] Cherry JD. 2004. The chronology of the 2002-2003 SARS mini pandemic. Paediatr Respir Rev 5:262–269. doi:10.1016/j.prrv.2004.07.009 15531249 PMC7106085

[3] Perlman S, Masters PS. 2021. Coronaviridae: The viruses and their replication, p 410–448. In Fields Virology: Emerging Viruses, 7e. Lippincott Williams & Wilkins, a Wolters Kluwer business.

[4] Zhou P, Yang X-L, Wang X-G, Hu B, Zhang L, Zhang W, Si H-R, Zhu Y, Li B, Huang C-L, et al.. 2020. A pneumonia outbreak associated with a new coronavirus of probable bat origin. Nature 579:270–273. doi:10.1038/s41586-020-2012-7 32015507 PMC7095418

[5] Wu Z, Han Y, Wang Y, Liu B, Zhao L, Zhang J, Su H, Zhao W, Liu L, Bai S, Dong J, Sun L, Zhu Y, Zhou S, Song Y, Sui H, Yang J, Wang J, Zhang S, Qian Z, Jin Q. 2023. A comprehensive survey of bat sarbecoviruses across China in relation to the origins of SARS-CoV and SARS-CoV-2. Natl Sci Rev 10:nwac213. doi:10.1093/nsr/nwac213 37425654 PMC10325003

[6] Ge X-Y, Li J-L, Yang X-L, Chmura AA, Zhu G, Epstein JH, Mazet JK, Hu B, Zhang W, Peng C, Zhang Y-J, Luo C-M, Tan B, Wang N, Zhu Y, Crameri G, Zhang S-Y, Wang L-F, Daszak P, Shi Z-L. 2013. Isolation and characterization of a bat SARS-like coronavirus that uses the ACE2 receptor. Nature 503:535–538. doi:10.1038/nature12711 24172901 PMC5389864

[7] Lam T-Y, Jia N, Zhang Y-W, Shum M-H, Jiang J-F, Zhu H-C, Tong Y-G, Shi Y-X, Ni X-B, Liao Y-S, et al.. 2020. Identifying SARS-CoV-2-related coronaviruses in Malayan pangolins. Nature 583:282–285. doi:10.1038/s41586-020-2169-0 32218527

[8] Cui X, Fan K, Liang X, Gong W, Chen W, He B, Chen X, Wang H, Wang X, Zhang P, et al.. 2023. Virus diversity, wildlife-domestic animal circulation and potential zoonotic viruses of small mammals, pangolins and zoo animals. Nat Commun 14:2488. doi:10.1038/s41467-023-38202-4 37120646 PMC10148632

[9] Liu P, Jiang J-Z, Wan X-F, Hua Y, Li L, Zhou J, Wang X, Hou F, Chen J, Zou J, Chen J. 2020. Are pangolins the intermediate host of the 2019 novel coronavirus (SARS-CoV-2)? PLoS Pathog 16:e1008421. doi:10.1371/journal.ppat.1008421 32407364 PMC7224457

[10] Xiao K, Zhai J, Feng Y, Zhou N, Zhang X, Zou J-J, Li N, Guo Y, Li X, Shen X, Zhang Z, Shu F, Huang W, Li Y, Zhang Z, Chen R-A, Wu Y-J, Peng S-M, Huang M, Xie W-J, Cai Q-H, Hou F-H, Chen W, Xiao L, Shen Y. 2020. Isolation of SARS-CoV-2-related coronavirus from Malayan pangolins. Nature 583:286–289. doi:10.1038/s41586-020-2313-x 32380510

[11] Sitam FT, Salgado-Lynn M, Denel A, Panjang E, McEwing R, Lightson A, Ogden R, Maruji NA, Yahya NK, Ngau C, Mohd Kulaimi NA, Ithnin H, Rovie-Ryan J, Abu Bakar MS, Ewart KM. 2023. Phylogeography of the Sunda pangolin, Manis javanica: implications for taxonomy, conservation management and wildlife forensics. Ecol Evol 13:e10373. doi:10.1002/ece3.10373 37593756 PMC10427774

[12] Delaune D, Hul V, Karlsson EA, Hassanin A, Ou TP, Baidaliuk A, Gámbaro F, Prot M, Tu VT, Chea S, Keatts L, Mazet J, Johnson CK, Buchy P, Dussart P, Goldstein T, Simon-Lorière E, Duong V. 2021. A novel SARS-CoV-2 related coronavirus in bats from Cambodia. Nat Commun 12:6563. doi:10.1038/s41467-021-26809-4 34753934 PMC8578604

[13] Wacharapluesadee S, Tan CW, Maneeorn P, Duengkae P, Zhu F, Joyjinda Y, Kaewpom T, Chia WN, Ampoot W, Lim BL, Worachotsueptrakun K, Chen V-W, Sirichan N, Ruchisrisarod C, Rodpan A, Noradechanon K, Phaichana T, Jantarat N, Thongnumchaima B, Tu C, Crameri G, Stokes MM, Hemachudha T, Wang L-F. 2021. Evidence for SARS-CoV-2 related coronaviruses circulating in bats and pangolins in Southeast Asia. Nat Commun 12:972. doi:10.1038/s41467-021-21240-1 33563978 PMC7873279

[14] Worthington BM, Wong PY-H, Kumaree KK, Prigge T-L, Ng KH, Liao Y, Martelli P, Churgin S, Lee FK, Perkins C, et al.. 2024. Serological evidence of sarbecovirus exposure along Sunda pangolin trafficking pathways. BMC Biol 22:274. doi:10.1186/s12915-024-02074-x 39593133 PMC11600613

[15] Shi W, Shi M, Que T-C, Cui X-M, Ye R-Z, Xia L-Y, Hou X, Zheng J-J, Jia N, Xie X, et al.. 2022. Trafficked Malayan pangolins contain viral pathogens of humans. Nat Microbiol 7:1259–1269. doi:10.1038/s41564-022-01181-1 35918420 PMC9352580

[16] Niu S, Wang J, Bai B, Wu L, Zheng A, Chen Q, Du P, Han P, Zhang Y, Jia Y, Qiao C, Qi J, Tian W-X, Wang H-W, Wang Q, Gao GF. 2021. Molecular basis of cross-species ACE2 interactions with SARS-CoV-2-like viruses of pangolin origin. EMBO J 40:e107786. doi:10.15252/embj.2021107786 34018203 PMC8209949

[17] Huang X-Y, Chen Q, Sun M-X, Zhou H-Y, Ye Q, Chen W, Peng J-Y, Qi Y-N, Zhai J-Q, Tian Y, Liu Z-X, Huang Y-J, Deng Y-Q, Li X-F, Wu A, Yang X, Yang G, Shen Y, Qin C-F. 2023. A pangolin-origin SARS-CoV-2-related coronavirus: infectivity, pathogenicity, and cross-protection by preexisting immunity. Cell Discov 9:59. doi:10.1038/s41421-023-00557-9 37330497 PMC10276878

[18] Hou YJ, Chiba S, Leist SR, Meganck RM, Martinez DR, Schäfer A, Catanzaro NJ, Sontake V, West A, Edwards CE, Yount B, Lee RE, Gallant SC, Zost SJ, Powers J, Adams L, Kong EF, Mattocks M, Tata A, Randell SH, Tata PR, Halfmann P, Crowe JE Jr, Kawaoka Y, Baric RS. 2023. Host range, transmissibility and antigenicity of a pangolin coronavirus. Nat Microbiol 8:1820–1833. doi:10.1038/s41564-023-01476-x 37749254 PMC10522490

[19] Lan J, Ge J, Yu J, Shan S, Zhou H, Fan S, Zhang Q, Shi X, Wang Q, Zhang L, Wang X. 2020. Structure of the SARS-CoV-2 spike receptor-binding domain bound to the ACE2 receptor. Nature 581:215–220. doi:10.1038/s41586-020-2180-5 32225176

[20] Chan JF-W, Huang X, Hu B, Chai Y, Shi H, Zhu T, Yuen TT-T, Liu Y, Liu H, Shi J, Wen L, Shuai H, Hou Y, Yoon C, Cai J-P, Zhang AJ, Zhou J, Yin F, Yuan S, Zhang B-Z, Brindley MA, Shi Z-L, Yuen K-Y, Chu H. 2023. Altered host protease determinants for SARS-CoV-2 Omicron. Sci Adv 9:eadd3867. doi:10.1126/sciadv.add3867 36662861 PMC9858505

[21] Cao Y, Yisimayi A, Jian F, Song W, Xiao T, Wang L, Du S, Wang J, Li Q, Chen X, et al.. 2022. BA.2.12.1, BA.4 and BA.5 escape antibodies elicited by Omicron infection. Nature 608:593–602. doi:10.1038/s41586-022-04980-y 35714668 PMC9385493

[22] Starr TN, Czudnochowski N, Liu Z, Zatta F, Park Y-J, Addetia A, Pinto D, Beltramello M, Hernandez P, Greaney AJ, et al.. 2021. SARS-CoV-2 RBD antibodies that maximize breadth and resistance to escape. Nature 597:97–102. doi:10.1038/s41586-021-03807-6 34261126 PMC9282883

[23] Ye G, Liu B, Li F. 2022. Cryo-EM structure of a SARS-CoV-2 omicron spike protein ectodomain. Nat Commun 13:1214. doi:10.1038/s41467-022-28882-9 35241675 PMC8894419

[24] Liu H, Wilson IA. 2022. Protective neutralizing epitopes in SARS-CoV-2. Immunol Rev 310:76–92. doi:10.1111/imr.13084 35599305 PMC9348472

[25] Dadonaite B, Brown J, McMahon TE, Farrell AG, Figgins MD, Asarnow D, Stewart C, Lee J, Logue J, Bedford T, Murrell B, Chu HY, Veesler D, Bloom JD. 2024. Spike deep mutational scanning helps predict success of SARS-CoV-2 clades. Nature 631:617–626. doi:10.1038/s41586-024-07636-1 38961298 PMC11254757

[26] Leman JK, Weitzner BD, Lewis SM, Adolf-Bryfogle J, Alam N, Alford RF, Aprahamian M, Baker D, Barlow KA, Barth P, et al.. 2020. Macromolecular modeling and design in Rosetta: recent methods and frameworks. Nat Methods 17:665–680. doi:10.1038/s41592-020-0848-2 32483333 PMC7603796

[27] Cao Y, Jian F, Wang J, Yu Y, Song W, Yisimayi A, Wang J, An R, Chen X, Zhang N, et al.. 2023. Imprinted SARS-CoV-2 humoral immunity induces convergent Omicron RBD evolution. Nature 614:521–529. doi:10.1038/s41586-022-05644-7 36535326 PMC9931576

[28] Wrobel AG, Benton DJ, Xu P, Calder LJ, Borg A, Roustan C, Martin SR, Rosenthal PB, Skehel JJ, Gamblin SJ. 2021. Structure and binding properties of Pangolin-CoV spike glycoprotein inform the evolution of SARS-CoV-2. Nat Commun 12:837. doi:10.1038/s41467-021-21006-9 33547281 PMC7864994

[29] Guo C, Yu Y, Liu J, Jian F, Yang S, Song W, Yu L, Shao F, Cao Y. 2025. Antigenic and virological characteristics of SARS-CoV-2 variants BA.3.2, XFG, and NB.1.8.1. Lancet Infect Dis 25:e374–e377. doi:10.1016/S1473-3099(25)00308-1 40484018

[30] Wu F, Zhao S, Yu B, Chen Y-M, Wang W, Song Z-G, Hu Y, Tao Z-W, Tian J-H, Pei Y-Y, Yuan M-L, Zhang Y-L, Dai F-H, Liu Y, Wang Q-M, Zheng J-J, Xu L, Holmes EC, Zhang Y-Z. 2020. A new coronavirus associated with human respiratory disease in China. Nature 579:265–269. doi:10.1038/s41586-020-2008-3 32015508 PMC7094943

[31] Cereghino C, Michalak K, DiGiuseppe S, Guerra J, Yu D, Faraji A, Sharp AK, Brown AM, Kang L, Weger-Lucarelli J, Michalak P. 2024. Evolution at Spike protein position 519 in SARS-CoV-2 facilitated adaptation to humans. Npj Viruses 2:29. doi:10.1038/s44298-024-00036-2 40295673 PMC11721114

[32] Dadonaite B, Harari S, Larsen BB, Kampman L, Harteloo A, Elias-Warren A, Chu HY, Bloom JD. 2025. Spike mutations that affect the function and antigenicity of recent KP.3.1.1-like SARS-CoV-2 variants. J Virol 99:e0142325. doi:10.1128/jvi.01423-25 41081510 PMC12614646

[33] Katoh K, Misawa K, Kuma K, Miyata T. 2002. MAFFT: a novel method for rapid multiple sequence alignment based on fast Fourier transform. Nucleic Acids Res 30:3059–3066. doi:10.1093/nar/gkf436 12136088 PMC135756

[34] Minh BQ, Schmidt HA, Chernomor O, Schrempf D, Woodhams MD, von Haeseler A, Lanfear R. 2020. IQ-TREE 2: new models and efficient methods for phylogenetic inference in the genomic era. Mol Biol Evol 37:1530–1534. doi:10.1093/molbev/msaa015 32011700 PMC7182206

[35] Hoang DT, Chernomor O, von Haeseler A, Minh BQ, Vinh LS. 2018. UFBoot2: improving the ultrafast bootstrap approximation. Mol Biol Evol 35:518–522. doi:10.1093/molbev/msx281 29077904 PMC5850222

[36] Kalyaanamoorthy S, Minh BQ, Wong TKF, von Haeseler A, Jermiin LS. 2017. ModelFinder: fast model selection for accurate phylogenetic estimates. Nat Methods 14:587–589. doi:10.1038/nmeth.4285 28481363 PMC5453245

[37] Niwa H, Yamamura K, Miyazaki J. 1991. Efficient selection for high-expression transfectants with a novel eukaryotic vector. Gene 108:193–199. doi:10.1016/0378-1119(91)90434-d 1660837

[38] Ferreira I, Kemp SA, Datir R, Saito A, Meng B, Rakshit P, Takaori-Kondo A, Kosugi Y, Uriu K, Kimura I, Shirakawa K, Abdullahi A, Agarwal A, Ozono S, Tokunaga K, Sato K, Gupta RK. 2021. CITIID-NIHR BioResource COVID-19 collaboration, Indian SARS-CoV-2 genomics consortium, genotype to phenotype Japan (G2P-Japan). Consortium:989–994. doi:10.1093/infdis/jiab368

[39] Ozono S, Zhang Y, Ode H, Sano K, Tan TS, Imai K, Miyoshi K, Kishigami S, Ueno T, Iwatani Y, Suzuki T, Tokunaga K. 2021. SARS-CoV-2 D614G spike mutation increases entry efficiency with enhanced ACE2-binding affinity. Nat Commun 12:848. doi:10.1038/s41467-021-21118-2 33558493 PMC7870668

[40] Kaku Y, Okumura K, Kawakubo S, Uriu K, Chen L, Kosugi Y, Uwamino Y, Begum MM, Leong S, Ikeda T, Sadamasu K, Asakura H, Nagashima M, Yoshimura K, Ito J, Sato K. 2024. Virological characteristics of the SARS-CoV-2 XEC variant. Lancet Infect Dis 24:e736. doi:10.1016/S1473-3099(24)00731-X 39521009

[41] Uriu K, Ito J, Kosugi Y, Tanaka YL, Mugita Y, Guo Z, Hinay AA Jr, Putri O, Kim Y, Shimizu R, Begum MM, Jonathan M, Saito A, Ikeda T, Sato K. 2023. Transmissibility, infectivity, and immune evasion of the SARS-CoV-2 BA.2.86 variant. Lancet Infect Dis 23:e460–e461. doi:10.1016/S1473-3099(23)00575-3 37734391

[42] Yajima H, Anraku Y, Kaku Y, Kimura KT, Plianchaisuk A, Okumura K, Nakada-Nakura Y, Atarashi Y, Hemmi T, Kuroda D, et al.. 2024. Structural basis for receptor-binding domain mobility of the spike in SARS-CoV-2 BA.2.86 and JN.1. Nat Commun 15:8574. doi:10.1038/s41467-024-52808-2 39375326 PMC11458767

[43] Kaku Y, Okumura K, Padilla-Blanco M, Kosugi Y, Uriu K, Hinay AA Jr, Chen L, Plianchaisuk A, Kobiyama K, Ishii KJ, Zahradnik J, Ito J, Sato K. 2024. Virological characteristics of the SARS-CoV-2 JN.1 variant. Lancet Infect Dis 24:e82. doi:10.1016/S1473-3099(23)00813-7 38184005

[44] Ozono S, Zhang Y, Tobiume M, Kishigami S, Tokunaga K. 2020. Super-rapid quantitation of the production of HIV-1 harboring a luminescent peptide tag. J Biol Chem 295:13023–13030. doi:10.1074/jbc.RA120.013887 32719008 PMC7489901

[45] Xu H, Wang B, Zhao T-N, Liang Z-T, Peng T-B, Song X-H, Wu J-J, Wang Y-C, Su X-D. 2021. Structure-based analyses of neutralization antibodies interacting with naturally occurring SARS-CoV-2 RBD variants. Cell Res 31:1126–1129. doi:10.1038/s41422-021-00554-1 34480123 PMC8413711

[46] Walls AC, Park Y-J, Tortorici MA, Wall A, McGuire AT, Veesler D. 2020. Structure, function, and antigenicity of the SARS-CoV-2 spike glycoprotein. Cell 181:281–292. doi:10.1016/j.cell.2020.02.058 32155444 PMC7102599

[47] Dong J, Zost SJ, Greaney AJ, Starr TN, Dingens AS, Chen EC, Chen RE, Case JB, Sutton RE, Gilchuk P, et al.. 2021. Genetic and structural basis for SARS-CoV-2 variant neutralization by a two-antibody cocktail. Nat Microbiol 6:1233–1244. doi:10.1038/s41564-021-00972-2 34548634 PMC8543371

[48] Nivón LG, Moretti R, Baker D. 2013. A Pareto-optimal refinement method for protein design scaffolds. PLoS One 8:e59004. doi:10.1371/journal.pone.0059004 23565140 PMC3614904

[49] Park H, Bradley P, Greisen P, Liu Y, Mulligan VK, Kim DE, Baker D, DiMaio F. 2016. Simultaneous optimization of biomolecular energy functions on features from small molecules and macromolecules. J Chem Theory Comput 12:6201–6212. doi:10.1021/acs.jctc.6b00819 27766851 PMC5515585

[50] Fujita S, Kosugi Y, Kimura I, Tokunaga K, Ito J, Sato K, Genotype to Phenotype Japan (G2P-Japan) Consortium. 2023. Determination of the factors responsible for the tropism of SARS-CoV-2-related bat coronaviruses to Rhinolophus bat ACE2. J Virol 97:e0099023. doi:10.1128/jvi.00990-23 37724881 PMC10779674

