# Supplementary material for: Genetic diversity of pangolin coronaviruses reveals a key immuno-evasive substitution at spike residue 519
Source: J Virol. 2026 Jun 10;100(7):e00352-26. doi: 10.1128/jvi.00352-26 (PMC13386858; doi:10.1128/jvi.00352-26)
Supplement: Supplemental figures — Fig. S1 and S2. [file jvi.00352-26-s0001.pdf]

Amino acid position

	285					290					300
SARS-CoV-2	I	T	D	A	V	D	C	A	L	D	P
GD/1/2019	I	T	D	A	V	D	C	A	L	D	P
cDNA8	I	T	D	A	V	D	C	A	L	D	P
cDNA9	I	T	D	A	V	D	C	A	L	D	P
cDNA16	I	T	D	A	V	E	C	A	L	D	P
cDNA18	I	T	D	A	V	D	C	A	L	D	P
cDNA20	I	T	D	A	V	D	C	A	L	D	P
cDNA31	I	T	D	A	V	D	C	A	L	D	P
GD/P79-9/2019	I	T	D	A	V	E	C	A	L	D	P
GD/M5-9/2019	I	T	D	A	V	D	C	A	L	D	P
MP789	I	T	D	A	V	D	C	A	L	D	P
GD/P44-9/2019	I	T	D	A	V	D	C	A	L	D	P

Amino acid position

	315					320					325
SARS-CoV-2	T	S	N	F	R	V	Q	P	T	E	S
GD/1/2019	T	S	N	F	R	V	Q	P	T	E	S
cDNA8	T	S	N	F	R	V	Q	P	T	E	S
cDNA9	T	S	N	F	R	V	Q	P	T	E	S
cDNA16	T	S	N	F	R	V	Q	P	T	E	S
cDNA18	T	S	N	F	R	V	Q	P	T	E	S
cDNA20	T	S	N	F	R	V	Q	P	T	E	S
cDNA31	T	S	N	F	R	V	Q	P	T	E	S
GD/P79-9/2019	T	S	N	F	R	V	Q	P	T	E	S
GD/M5-9/2019	T	S	N	F	R	A	Q	P	T	E	S
MP789	T	S	N	F	R	V	Q	P	T	E	S
GD/P44-9/2019	T	S	N	F	R	X	Q	P	T	E	S

Amino acid position

	515					520					525
SARS-CoV-2	F	E	L	L	H	A	P	A	T	V	C
GD/1/2019	F	E	L	L	N	A	P	A	T	V	C
cDNA8	F	E	L	L	N	A	P	A	T	V	C
cDNA9	F	E	L	L	N	A	P	A	T	V	C
cDNA16	F	E	L	L	N	A	P	A	T	V	C
cDNA18	F	E	L	L	N	A	P	A	T	V	C
cDNA20	F	E	L	L	N	A	P	A	T	V	C
cDNA31	F	E	L	L	N	A	P	A	T	V	C
GD/P79-9/2019	F	E	L	L	N	A	P	A	T	V	C
GD/M5-9/2019	F	E	L	L	N	A	P	A	T	V	C
MP789	F	E	L	L	K	A	P	A	T	V	C
GD/P44-9/2019	F	E	L	L	K	A	P	A	T	V	C

Amino acid position

	630					635					640
SARS-CoV-2	T	P	T	W	R	V	Y	S	T	G	S
GD/1/2019	T	P	T	W	S	V	Y	S	T	G	S
cDNA8	T	P	T	W	R	V	Y	S	T	G	S
cDNA9	T	P	T	W	R	V	Y	S	T	G	S
cDNA16	T	P	T	W	R	V	Y	S	T	G	S
cDNA18	T	P	T	W	S	V	Y	S	T	G	S
cDNA20	T	P	T	W	R	V	Y	S	T	G	S
cDNA31	T	P	T	W	R	V	Y	S	T	G	S
GD/P79-9/2019	T	P	T	W	R	V	Y	S	T	G	S
GD/M5-9/2019	T	P	T	W	R	V	Y	S	T	G	S
MP789	T	P	T	W	R	V	Y	S	T	G	S
GD/P44-9/2019	T	P	T	W	R	V	Y	S	T	G	S

Amino acid position

	655					660					665
SARS-CoV-2	H	V	N	N	S	Y	E	C	D	I	P
GD/1/2019	H	V	N	N	S	Y	E	C	D	I	P
cDNA8	H	V	N	N	T	Y	E	C	D	I	P
cDNA9	H	V	N	N	T	Y	E	C	D	I	P
cDNA16	H	V	N	N	T	Y	E	C	D	I	P
cDNA18	H	V	N	N	S	Y	E	C	D	I	P
cDNA20	H	V	N	N	T	Y	E	C	D	I	P
cDNA31	H	V	N	N	T	Y	E	C	D	I	P
GD/P79-9/2019	H	V	N	N	T	Y	E	C	D	I	P
GD/M5-9/2019	H	V	N	N	T	Y	E	C	D	I	P
MP789	H	V	N	N	T	Y	E	C	D	I	P
GD/P44-9/2019	H	V	N	N	T	Y	E	C	D	I	P

Fig. S1 Amino acid sequence alignment of SARS-CoV-2 and GD pCoVs. The amino acid residues between residues 285 to 300, 315 to 325, 515 to 525, 630 to 640, and 655 to 665 in SARS-CoV-2 and GD pCoVs. The residues different among pCoVs positioned at 290, 320, 519 and 634 are indicated.

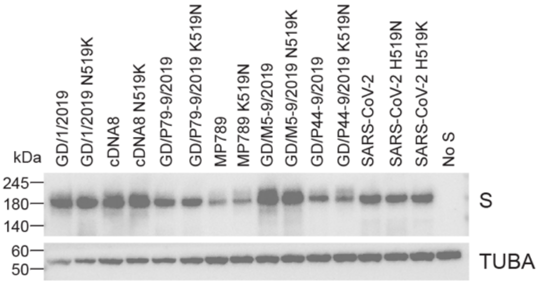

Fig. S2 Western bl**otting of S protein in pseudovirus-expressing cells.** Representative blots of S-expressing cells are shown. TUBA is an internal control for the cells. kDa, kilodalton.
