# Supplementary material for: Genetic diversity of pangolin coronaviruses reveals a key immuno-evasive substitution at spike residue 519
Source: J Virol. 2026 Jun 10;100(7):e00352-26. doi: 10.1128/jvi.00352-26 (PMC13386858; doi:10.1128/jvi.00352-26)
Supplement: Table S1 — Sequence data and accession numbers used in this study. [file jvi.00352-26-s0002.docx]

**Table S1. Sequence data and accession numbers used in this study.**

| **Isolate Name** | **Definition** | **Accession Number** | **Host** |
| --- | --- | --- | --- |
| WIV1 | Bat SARS-like coronavirus WIV1, complete genome | KF367457.1 | *Rhinolophus sinicus* |
| SARS-CoV-2 | Severe acute respiratory syndrome coronavirus 2 isolate Wuhan-Hu-1, complete genome | NC_045512.2 | *Homo sapiens* |
| RaTG13 | Bat coronavirus RaTG13, complete genome | MN996532.2 | *Rhinolophus affinis* |
| BANAL-20-236 | Bat coronavirus isolate BANAL-20-236/Laos/2020, complete genome | MZ937003.2 | *Rhinolophus marshalli* |
| BANAL-20-52 | Bat coronavirus isolate BANAL-20-52/Laos/2020, complete genome | MZ937000.1 | *Rhinolophus malayanus* |
| PCoV_GX-P5L | Pangolin coronavirus isolate PCoV_GX-P5L, complete genome | MT040335.1 | *Manis javanica* |
| LYRa11 | Rhinolophus affinis coronavirus isolate LYRa11, complete genome | KF569996.1 | *Rhinolophus affinis* |
| PCoV_GX-P4L | Pangolin coronavirus isolate PCoV_GX-P4L, complete genome | MT040333.1 | *Manis javanica* |
| PCoV_GX-P5E | Pangolin coronavirus isolate PCoV_GX-P5E, complete genome | MT040336.1 | *Manis javanica* |
| PCoV_GX-P1E | Pangolin coronavirus isolate PCoV_GX-P1E, complete genome | MT040334.1 | *Manis javanica* |
| PCoV_GX-P2V | Pangolin coronavirus isolate PCoV_GX-P2V, complete genome | MT072864.1 | *Manis javanica* |
| GD/P79-9/2019 | MAG: Pangolin coronavirus isolate GD/P79-9/2019, complete genome | OQ297708.1 | *Manis javanica* |
| Tor2 | SARS coronavirus Tor2, complete genome | NC_004718.3 | *Homo sapiens* |
| Rc-o319 | Severe acute respiratory syndrome-related coronavirus Rc-o319 RNA, complete genome | LC556375.1 | *Rhinolophus cornutus* |
| Rc-kw8 | Sarbecovirus sp. Rc-kw8 RNA, complete genome | LC663793.1 | *Rhinolophus cornutus* |
| GD/M5-9/2019 | MAG: Pangolin coronavirus isolate GD/M5-9/2019, partial genome | OQ297700.1 | *Manis javanica* |
| BANAL-20-103 | Bat coronavirus isolate BANAL-20-103/Laos/2020, complete genome | MZ937001.1 | *Rhinolophus pusillus* |
| GD/P44-9/2019 | MAG: Pangolin coronavirus isolate GD/P44-9/2019, partial genome | OQ297707.1 | *Manis javanica* |
| MP789 | Pangolin coronavirus isolate MP789, complete genome | MT121216.1 | *Manis javanica* |
| Rc-os20 | Sarbecovirus sp. Rc-os20 RNA, complete genome | LC663958.1 | *Rhinolophus cornutus* |
| Rc-mk2 | Sarbecovirus sp. Rc-mk2 RNA, complete genome | LC663959.1 | *Rhinolophus cornutus* |
| cDNA8-S | Pangolin coronavirus isolate cDNA8-S surface glycoprotein (S) gene, complete cds | MT799521.1 | *Manis javanica* |
| cDNA9-S | Pangolin coronavirus isolate cDNA9-S surface glycoprotein (S) gene, complete cds | MT799522.1 | *Manis javanica* |
| cDNA16-S | Pangolin coronavirus isolate cDNA16-S surface glycoprotein (S) gene, complete cds | MT799523.1 | *Manis javanica* |
| cDNA18-S | Pangolin coronavirus isolate cDNA18-S surface glycoprotein (S) gene, complete cds | MT799524.1 | *Manis javanica* |
| cDNA20-S | Pangolin coronavirus isolate cDNA20-S surface glycoprotein (S) gene, complete cds | MT799525.1 | *Manis javanica* |
| cDNA31-S | Pangolin coronavirus isolate cDNA31-S surface glycoprotein (S) gene, complete cds | MT799526.1 | *Manis javanica* |
| GD/1/2019 | BetaCoV/pangolin/Guangdong/1/2019 | EPI-ISL-410721 | *Manis javanica* |
| Ra22QT106 | Horseshoe bat sarbecovirus isolate Ra22QT106, complete genome | OR233322.1 | *Rhinolophus affinis* |
| Ra22QT135 | Horseshoe bat sarbecovirus isolate Ra22QT135, complete genome | OR233323.1 | *Rhinolophus affinis* |
| Ra22QT77 | Horseshoe bat sarbecovirus isolate Ra22QT77, complete genome | OR233324.1 | *Rhinolophus affinis* |
| Ra22QT137 | Horseshoe bat sarbecovirus isolate Ra22QT137, complete genome | OR233328.1 | *Rhinolophus affinis* |
| Rp22DB159 | Horseshoe bat sarbecovirus isolate Rp22DB159, complete genome | OR233302.1 | *Rhinolophus pusillus* |
| BtSY2 | Bat SARS-like virus BtSY2, complete cds. | OP963576.1 | *Rhinolophus marshalli* |
