# Supplementary material for: Genetic diversity of pangolin coronaviruses reveals a key immuno-evasive substitution at spike residue 519
Source: J Virol. 2026 Jun 10;100(7):e00352-26. doi: 10.1128/jvi.00352-26 (PMC13386858; doi:10.1128/jvi.00352-26)
Supplement: Table S2 — Primers used in this study. [file jvi.00352-26-s0003.docx]

**Table S2. Primers used in this study.**

| **Primer Name** | **Primer sequence (5' to 3')** | **Purpose** |
| --- | --- | --- |
| pC-opt-GD/1/2019+MP789+GD/P44/9/2019-Fw | CTATAGGGCGAATTGGGTACCATGCTGTTCTTCTTC | Preparation of S expression plasmid |
| pC-opt-GD/1/2019-inf-Rv | AGCTCCACCGCGGTGGCGGCCGCTCAAGTATAGTGCAGTTT | Preparation of S expression plasmid |
| pC-opt-MP789+GD/P44/9/2019-Rv | GAGCTCCACCGCGGTGGCGGCCGCTCATGTATAGTGCAG | Preparation of S expression plasmid |
| pC-opt-cDNA8+GD/M5-9/2019-Fw | CTATAGGGCGAATTGGGTACCATGCTCTTCTTCTTC | Preparation of S expression plasmid |
| pC-opt-GD/P79-9/2019-Fw | CTATAGGGCGAATTGGGTACCATGCTGTTTTTTTTC | Preparation of S expression plasmid |
| pC-opt-cDNA8+GD/P79-9/2019+GD/M5-9/2019-Rv | GAGCTCCACCGCGGTGGCGGCCGCTCAGGTGTAGTGCAG | Preparation of S expression plasmid |
| pC-opt-SARS-CoV2-Rv | CCACCGCGGTGGCGGCCGCTCTAGATTCAGGTG | Preparation of S expression plasmid |
| pC-opt-SARS-CoV2-Fw | CTATAGGGCGAATTGGGTACCATGTTTGTGTTC | Preparation of S expression plasmid |
| GD/1/2019-N519K-Rv | CACTGTGGCTGGGGCCTTCAGCAGTTCAAAGGA | Preparation of S expression plasmid |
| GD/1/2019-N519K-Fw | TCCTTTGAACTGCTGAAGGCCCCAGCCACAGTG | Preparation of S expression plasmid |
| cDNA8+GD/M5-9/2019-N519K-Rv | GACGGTAGCGGGAGCCTTCAACAGTTCAAAACT | Preparation of S expression plasmid |
| cDNA8+GD/M5-9/2019-N519K-Fw | AGTTTTGAACTGTTGAAGGCTCCCGCTACCGTC | Preparation of S expression plasmid |
| GD/P79-9/2019-N519K-Rv | AACTGTGGCAGGAGCCTTGAGCAGTTCGAAGGA | Preparation of S expression plasmid |
| GD/P79-9/2019-N519K-Fw | TCCTTCGAACTGCTCAAGGCTCCTGCCACAGTT | Preparation of S expression plasmid |
| MP789+GD/P44-9/2019-K519N-Rv | GACGGTTGCGGGCGCGTTGAGAAGCTCGAAACT | Preparation of S expression plasmid |
| MP789+GD/P44-9/2019-K519N-Fw | AGTTTCGAGCTTCTCAACGCGCCCGCAACCGTC | Preparation of S expression plasmid |
| SARS-CoV2-H519N-Rv | CACTGTGGCAGGGGCGTTGAGCAGTTCAAAGGA | Preparation of S expression plasmid |
| SARS-CoV2-H519N-Fw | TCCTTTGAACTGCTCAACGCCCCTGCCACAGTG | Preparation of S expression plasmid |
| SARS-CoV2-H519K-Rv | CACTGTGGCAGGGGCCTTGAGCAGTTCAAAGGA | Preparation of S expression plasmid |
| SARS-CoV2-H519K-Fw | TCCTTTGAACTGCTCAAGGCCCCTGCCACAGTG | Preparation of S expression plasmid |
