# Supplementary material for: Genetic diversity of pangolin coronaviruses reveals a key immuno-evasive substitution at spike residue 519
Source: J Virol. 2026 Jun 10;100(7):e00352-26. doi: 10.1128/jvi.00352-26 (PMC13386858; doi:10.1128/jvi.00352-26)
Supplement: Table S3 — Human three-dose Pfizer-BioNTech vaccine sera used in this study. [file jvi.00352-26-s0004.docx]

**Table S3. Human 3-dose Pfizer-BioNTech vaccine sera used in this study.**

| **Donor  ID** | **Sex** | **Age** | **Date of  1st vaccination (YYYY-MM-DD)** | **Date of  2nd vaccination (YYYY-MM-DD)** | **Date of  3rd vaccination (YYYY-MM-DD)** | **Date of sampling after 3rd vaccination (YYYY-MM-DD)** | **Time interval between  3rd vaccination and sampling (days)** | **Prior  SARS-CoV-2  infection?** |
| --- | --- | --- | --- | --- | --- | --- | --- | --- |
| 5 | Female | 34 | 2021-04-09 | 2021-04-30 | 2022-01-22 | 2022-02-24 | 33 | No |
| 10 | Female | 53 | 2021-04-05 | 2021-04-26 | 2022-01-22 | 2022-02-14 | 23 | No |
| 27 | Male | 41 | 2021-04-06 | 2021-04-27 | 2022-01-22 | 2022-02-24 | 33 | No |
| 36 | Male | 34 | 2021-04-07 | 2021-04-27 | 2022-01-25 | 2022-02-25 | 31 | No |
| 41 | Female | 33 | 2021-04-08 | 2021-04-27 | 2022-01-26 | 2022-02-16 | 21 | No |
| 61 | Female | 48 | 2021-05-14 | 2021-06-04 | 2022-01-29 | 2022-02-14 | 16 | No |
| 66 | Male | 29 | 2021-03-05 | 2021-03-26 | 2022-01-24 | 2022-02-14 | 21 | No |
| 105 | Male | 49 | 2021-05-10 | 2021-06-07 | 2022-01-24 | 2022-02-15 | 22 | No |
| 109 | Female | 46 | 2021-05-14 | 2021-06-04 | 2022-01-26 | 2022-02-18 | 23 | No |
| 113 | Female | 32 | 2021-05-13 | 2021-06-04 | 2022-01-28 | 2022-02-18 | 21 | No |
| 116 | Female | 30 | 2021-05-18 | 2021-06-09 | 2022-01-25 | 2022-02-17 | 23 | No |
